# Large-scale comparison of machine learning methods for profiling prediction of kinase inhibitors

**DOI:** 10.1186/s13321-023-00799-5

**Published:** 2024-01-30

**Authors:** Jiangxia Wu, Yihao Chen, Jingxing Wu, Duancheng Zhao, Jindi Huang, MuJie Lin, Ling Wang

**Affiliations:** Guangdong Provincial Key Laboratory of Fermentation and Enzyme Engineering, Joint International Research Laboratory of Synthetic Biology and Medicine, Guangdong Provincial Engineering and Technology Research Center of Biopharmaceuticals, School of Biology and Biological Engineering, South China University of Technology, Guangzhou, 510006 China

**Keywords:** Kinase profiling, Machine learning, Deep learning, Molecular fingerprints, Molecular graphs

## Abstract

Conventional machine learning (ML) and deep learning (DL) play a key role in the selectivity prediction of kinase inhibitors. A number of models based on available datasets can be used to predict the kinase profile of compounds, but there is still controversy about the advantages and disadvantages of ML and DL for such tasks. In this study, we constructed a comprehensive benchmark dataset of kinase inhibitors, involving in 141,086 unique compounds and 216,823 well-defined bioassay data points for 354 kinases. We then systematically compared the performance of 12 ML and DL methods on the kinase profiling prediction task. Extensive experimental results reveal that (1) Descriptor-based ML models generally slightly outperform fingerprint-based ML models in terms of predictive performance. RF as an ensemble learning approach displays the overall best predictive performance. (2) Single-task graph-based DL models are generally inferior to conventional descriptor- and fingerprint-based ML models, however, the corresponding multi-task models generally improves the average accuracy of kinase profile prediction. For example, the multi-task FP-GNN model outperforms the conventional descriptor- and fingerprint-based ML models with an average AUC of 0.807. (3) Fusion models based on voting and stacking methods can further improve the performance of the kinase profiling prediction task, specifically, RF::AtomPairs + FP2 + RDKitDes fusion model performs best with the highest average AUC value of 0.825 on the test sets. These findings provide useful information for guiding choices of the ML and DL methods for the kinase profiling prediction tasks. Finally, an online platform called KIPP (https://kipp.idruglab.cn) and python software are developed based on the best models to support the kinase profiling prediction, as well as various kinase inhibitor identification tasks including virtual screening, compound repositioning and target fishing.

**Supplementary Information:**

The online version contains supplementary material available at 10.1186/s13321-023-00799-5.

## Introduction

The human kinome comprises more than 500 kinases, constituting approximately 1.7% of all human genes [1]. Protein kinases (PKs) play central roles in mediating most signaling pathways involved in cellular metabolism, transcription, cell cycle, apoptosis, and differentiation. Therefore, PKs have become one of the most interesting classes of drug targets for various diseases, including cancers [2–4], inflammation [5, 6], central nervous system disorders [7], cardiovascular diseases [8], complications of diabetes [9], and Alzheimer’s disease [10]. As such a significant class of targets, kinase inhibitors have been the focus of drug discovery. There are currently 71 FDA-approved small-molecule kinase inhibitors. In addition, approximately 110 innovative kinases are emerging as targets for drugs development in clinical trials [11]. Most FDA-approved drugs (63/71) targeting kinases are ATP-competitive inhibitors which inhibit kinases activity by binding to the ATP binding site of the kinase domain. However, the intrinsically highly conserved ATP binding sites of kinases may lead to off-target effects (i.e., low selectivity) of kinase inhibitors, potentially leading to undesirable side effects. Accordingly, identifying selective PK inhibitors remains an important challenge in the development of kinase-targeted drugs. Traditional kinase inhibitor assays are low-throughput methods that primarily measure the ability of compounds to reduce the phosphorylation activity for a given kinase (e.g. IC_50_) or their binding affinities to a kinase (dissociation constant, such as *K*_*i*_ and *K*_*d*_). Notably, such measurement methods typically do not extend to the ability of a compound to inhibit the entire kinome. High-throughput kinase profiling assay has also become feasible in recent years, but the excessive cost makes it difficult to use as a routine early stage of drug discovery efforts [12].

Based on experimental data, a number of computational methods have been developed and published elsewhere, aiming to significantly reduce the cost, time and laborious involved in experimental identification. Generally, these computational methods can be classified into two major categories: structure- and ligand-based kinase inhibition and/or profiling prediction approaches (called virtual assay). Molecular docking, commonly used in structure-based prediction methods for kinase inhibition, has good generalizability, but its accuracy depends on the crystal structure of the kinase and the accuracy of the scoring function [13, 14]. Ligand-based methods include pharmacophore modelling, and quantitative structure–activity relationship (QSAR) [15–21]. Based on different kinase inhibitors-associated datasets, ML and DL algorithms such as naive Bayesian (NB) [22–24], k-nearest neighbors (KNN) [24–26], random forest (RF) [27–30], support vector machine (SVM) [25, 26, 31], and deep neural network (DNN) [32, 33] have been used to construct models on the basis of various molecular descriptors and fingerprints for predicting a larger spectrum of kinases inhibition activities for a molecule. These established models play a key role in the theoretical prediction of kinase profiling due to their accuracy and speed of prediction results, and have accelerated the identification and optimization of kinase inhibitors in the early stage of drug discovery.

However, the existing kinase profiling models have the following shortcomings. Firstly, there are two major flaws in the modelling dataset for the kinase profiling prediction task. For one thing, the number of kinases involved in constructing the kinase profiling prediction models is small, limiting its versatility (narrow kinome prediction) compared to the human kinome containing more than 500. For example, the kinase profiling prediction models proposed by Bora and coworkers only includes 107 kinases [29, 34]. For another, the number of compounds in dataset are relatively small, which may lead to the limited generalization ability of the established models. For example, in 2020, Li et al. [34] proposed a virtual kinase profiling model against a panel of 391 kinases, however, there are approximately 40 kinases with less than 10 compounds (actives and inactives). Apparently, the predictive models based on these insufficient compound datasets may not achieve good generalization performance. Secondly, for different tailored modelling datasets, the existing models are constructed based on a specific molecular representation (i.e. molecular descriptors or fingerprints) by using only single or limited ML methods. Obviously, this lack of combined screening of molecular features and ML algorithms will result in the built models that may not be able to achieve the highest accuracy. In other words, it is impossible to assess which ML methods can achieve higher performance in building kinase profiling models from the existing studies. Thirdly, most of the existing kinase profiling predictive models are trained using conventional ML (e.g., KNN, NB, SVM and RF) algorithms, hile the advanced DL (especially graph neural network, GNN) algorithms, which have been successfully used to predict molecular properties and bioactivities, have seldom conducted for the kinase profiling prediction [35–38]. In addition, the reported kinase profiling predictive models have not been integrated into easy-to-use tools (e.g., local software package or online platform), which limits the use of these models by experts and non-experts in the field.

**Fig. 1:**
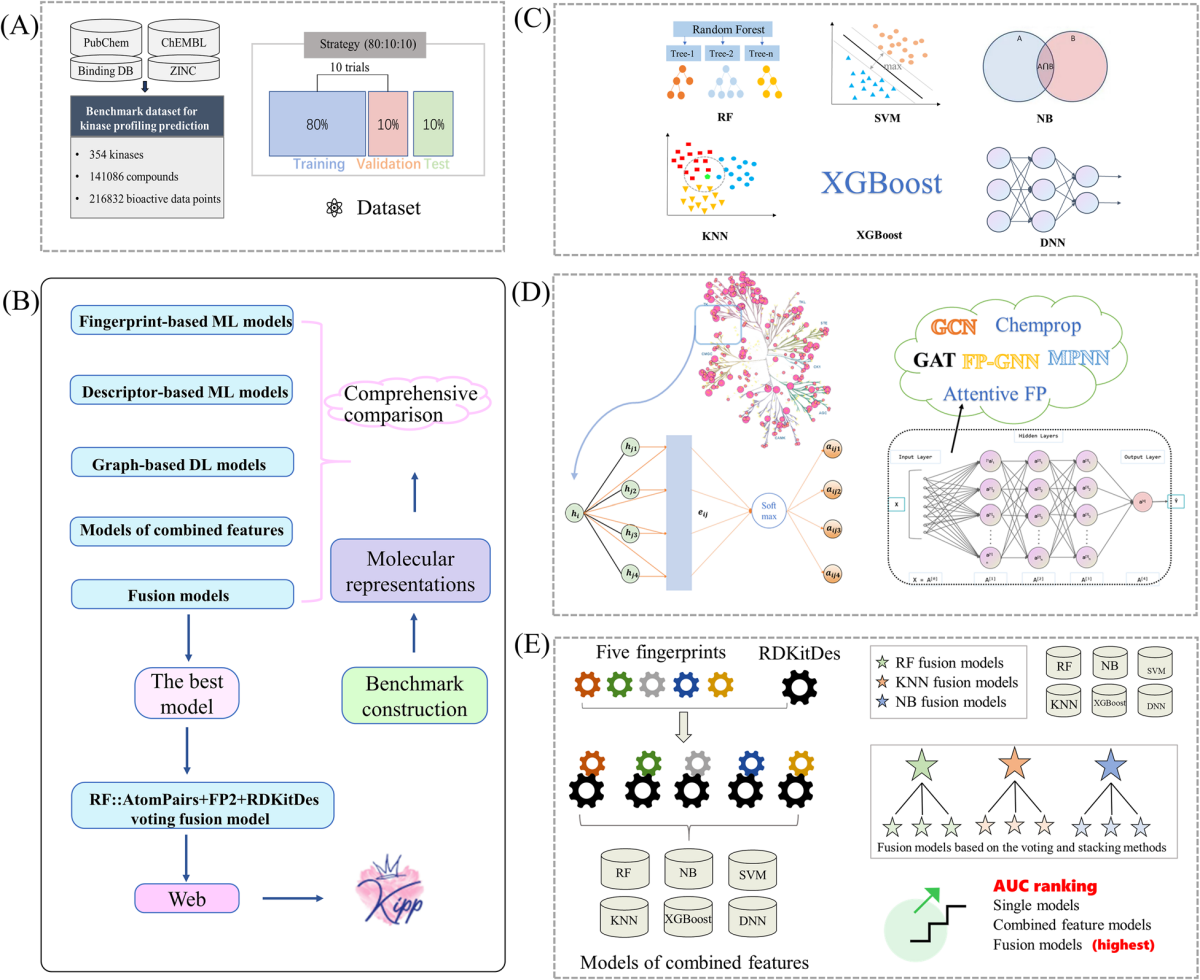
The scheme and workflow of this work. **A** Dataset collection. **B** Models construction and selecting the optimal model for the kinase profiling prediction task. **C** ML methods for the construction of fingerprint- and RDKitDes-based models. **D** DL methods for the construction of graph-based models. (E) Combined-features- and fusion-based models construction

To address the above-mentioned shortcomings regarding the kinase profiling prediction task, herein, we constructed a comprehensive kinase profiling prediction benchmark dataset (called KinaseNet) from multiple sources for 354 kinases. A total of 136,290 predictive models were then built based on three types of molecular representations (i.e. a set of molecular descriptors, five different molecular fingerprints, and molecular graphs) using five mainstream ML methods (e.g., KNN [39], NB [40], SVM [41], RF [42], and XGBoost [43]) and seven advanced DL algorithms including DNN [44], graph convolutional network (GCN) [45], graph attention network (GAT) [46], message passing neural networks (MPNN) [47], Attentive FP [48], D-MPNN (Chemprop) [49] and FP-GNN [50]. The performances of these ML and DL models were comprehensively compared and evaluated. The influences of the sizes of the modelling datasets and features selection on the performances of the kinase profiling models are also explored. Finally, the best models based on the comprehensive comparison results were used to develop an online platform and its python software for supporting kinase inhibitor drug discovery related tasks. The scheme and workflow of this work are shown in Fig. 1.

## Materials and methods

### Benchmark dataset for kinase profiling prediction

All quantitative compound-kinase associations were collected from ChEMBL (Version 29) [51], PubChem [52], BindingDB [53], and Zinc [54]. We then processed the raw data using the following steps: (1) only ATP-competitive kinase inhibition assay data (assay type: B) for each compound was kept, and compounds with detailed biological activities recorded as IC_50_, EC_50_, *K*_d_, or *K*_i_ were maintained; (2) the bioactivity units (g/mL, M, and nM) were translated to the standard unit in μM, molecules whose labels could not be unequivocally assigned (e.g., IC_50_, EC_50_, *K*_i_, or *K*_d_ < 100 or > 1 μM) were excluded; and if a compound has multiple inhibitory activity test data for a kinase, we averaged the reported bioactivity records as the final inhibitory activity value; (3) all molecular structures in the kinase profiling dataset were processed using the Standardizer package (https://github.com/flatkinson/standardiser, version 0.1.9), including removal of counter ions, solvent fractions and salts, and adding hydrogen atoms, and once all molecules were standardized, those with molecular weight greater than 1000 Da as well as duplicated molecules were removed; (4) compounds were labeled as actives (p*K*_i_/p*K*_d_/pIC_50_/pEC_50_ ≥ 6) and inactives (p*K*_i_/p*K*_d_/pIC_50_/pEC_50_ < 6) in each kinase [34, 55], and we preserved compound − kinase associations only for those kinases with at least 20 active molecules. After applying those criteria, the final comprehensive kinase profiling dataset consists of 141,086 molecules with 216,823 bioactive data points for 354 kinases. Each kinase dataset was randomly divided into three sub-datasets: training set (80%), validation set (10%), and test set (10%). The modelling datasets utilized in the present study are freely available at https://kipp.idruglab.cn/about.

### Molecular representations calculation

In this study, five molecular fingerprints including Morgan fingerprints (ECFP-like, 1024-bits) [56], MACCS keys (166-bits) [57], AtomParis fingerprints (1024-bits) [58], FP2 fingerprints (1024-bits) [59] and 2D pharmacophore fingerprints (PharmacoPFP, 38-bits) [60] were used to construct fingerprint-based predictive models. A set of 208 RDKit molecular descriptors (termed RDKitDes) was chosen for the development of descriptor-based predictive models. The fingerprints and descriptors were calculated using open source RDKit software (http://www.rdkit.org/, version: 2020.03.1).

In a molecular graph, the atomic and atomic pair features are used together as a feature matrix [61]. Chemprop and FP-GNN utilize RDKit software (version: 2020.09.5) to calculate molecular graphs. Other molecular graph-based representations were generated using DeepChem (version: 2.5.0). For example, the MolGraphConvFeatureizer module was used to calculate the molecular graphs for the GAT, MPNN, and Attentive FP models, while the ConvMolFeaturizer [62] module was used to compute the molecular graph representation for GCN models.

### Selection of ML and DL algorithms for the assessment and model construction

Five mainstream ML and seven advanced DL algorithms were used to build the kinase profiling predictive modes for 354 kinases. These modelling methods (Table 1) are briefly introduced as follows.

**Table 1:** Detailed ML and DL modelling methods used in this study

Method	Molecular feature	Hyperparameter optimization	Website
RF^*a*^	RDKitDES or fingerprints (Morgan, MACCS, AtomPairs, FP2, and PharmacoPFP)	Grid search	https://github.com/scikit-learn/scikit-learn
NB^*b*^		Grid search	https://github.com/scikit-learn/scikit-learn
SVM^*c*^		Grid search	https://github.com/scikit-learn/scikit-learn
KNN^*d*^		Grid search	https://github.com/scikit-learn/scikit-learn
XGBoost^*e*^		Grid search	https://github.com/dmlc/xgboost
DNN^*f*^		Grid search	https://deepchem.io/
GCN^*g*^	molecular graphs	Grid search	https://deepchem.io/
GAT^*h*^	molecular graphs	Grid search	https://deepchem.io/
MPNN^*i*^	molecular graphs	Grid search	https://deepchem.io/
Attentive FP^*j*^	molecular graphs	Grid search	https://deepchem.io/
Chemprop^*k*^	molecular graphs	Bayesian Optimization	https://github.com/chemprop/chemprop
FP-GNN^*l*^	molecular graphs and fixed molecular fingerprints (MACCS, PubChem, and Pharmacophore ErG fingerprints)	Bayesian optimization	https://github.com/idrugLab/FP-GNN

^*a*^ RF: Random forest

^*b*^ NB: Naïve Bayesian

^*c*^ SVM: Support vector machine

^*d*^ KNN: K-Nearest Neighbor

^*e*^ XGBoost: Extreme gradient boosting

^*f*^ DNN: Deep neural networks

^*g*^ GCN: Graph convolutional network

^*h*^ GAT: Graph attention network

^*i*^ MPNN: Message passing neural networks

^j^ Attentive FP

^k^ Chemprop: D-MPN

^*l*^ FP-GNN

#### Random forest (RF)

RF, developed by Svetnik et al.[42], is an ensemble recursive partitioning approach in which each recursive partitioning ‘tree’ is built from a bootstrapped sample of compounds, and each branch of a tree uses a random subset of descriptors [27]. The following five hyperparameters were tuned to achieve the optimal RF model: n_estimators (10–500), criterion (‘*gini’* and ‘*entropy’*), max_depth (0–15), min_samples_leaf (1–10), and max_features (‘*log2’*, ‘*auto’* and ‘*sqrt’*).

#### Naïve Bayesian (NB)

NB classifier is developed based on Bayes’ theorem [40] and widely used in molecular properties prediction and virtual screening (VS) projects [63–66]. Two hyperparameters were optimized for NB models construction: alpha (0.01–1) and binarize (0, 0.5, 0.8).

#### Support vector machine (SVM)

SVM was formally developed in 1995 [41] and quickly became a mainstream ML method due to its excellent performance in text classification tasks [67]. The principle of SVM is to determine the optimal hyperplane in the feature space by maximizing the boundaries between classes in *N*-dimensional space, which can distinguish objects with various class labels. Two hyperparmeters, Kernel coefficient (gamma, ‘auto’, 0.1–0.2) and penalty parameter C of the error term (C, from 1 to 100), were optimized for the development of SVM models.

#### K-nearest neighbor (KNN)

KNN is a commonly used supervised learning method with a simple mechanism. For a given test sample, it finds the *k* closest training samples in the training set based on distance measures (e.g., Manhattan, Euclidean, and Jaccard distance), and then makes a prediction based on the information of these *k* ‘neighbors’ [39]. In the training of KNN models, the default Euclidean distance metric was utilized, and three hyperparameters including n_neighbors (1–5), p (1–2), and weight function (‘uniform’, ‘distance’), were optimized.

#### Extreme gradient boosting (XGBoost)

XGBoost is one of the most representative ensemble ML algorithms under the gradient boosting framework [43]. It has been shown to achieve state-of-the-art (SOTA) performance on many standard classification benchmark datasets [37, 68, 69]. Seven hyperparameters were optimized: learning_rate (0.01–0.1), n_estimators (50–100), max_depth (3–5), min_child_weight (1–3), gamma (0–0.1), subsample (0.8–1.0), and colsample bytree (0.8–1.0).

#### Deep neural networks (DNN)

DNN is essentially an artificial neural network with an input layer, an output layer, and multiple hidden layers, which mimics the behavior of biological neural networks [44]. DNN consists of a large number of individual neurons [70, 71], and each neuron in the DNN architecture collects information from its associated neurons and a non-linear activation function was then used to activate the aggregated information. Three hyperparameters were optimized: dropouts (0.1, 0.2, 0.5), layer_sizes (64, 128, 256, 512) and weight_decay_penalty (0.01, 0.001, 0.0001).

#### Graph convolutional network (GCN)

GCN uses graph-structured data as features input [45], and consists of graph convolution layers, a readout layer, fully linked layers, and an output layer. The basic principle of GCN is to use edge information to aggregate node information, resulting in a new node representation. Several frameworks of GCN and variants have been proposed so far. For example, Duvenaud et al. [62] proposed a convolutional neural network that operates directly on molecular graphs, allowing end-to-end learning of prediction pipelines to exhibit better predictive performance for molecular property prediction tasks. Here, this GCN architecture was used to establish GCN models, and the following hyperparameters were optimized: weight decay (0, 10e-8, 10e-6, 10e-4), graph conv layers ([64, 64], [128, 128], [256, 256], learning rate (0.01, 0.001, 0.0001), and dense layer size (64, 128, 256).

#### Graph attention network (GAT)

GAT introduces an attention mechanism based on the GCN [46], which calculates the weights of the features of nodes and adjacent nodes through aggregation, and follows a self-aggregation strategy. GAT can better extract the spatial feature relationships of nodes compared to the GCN in the application of directed graphs [72]. Six hyperparameters were optimized in the training of the GAT models, including weight_decay (0, 10e-8, 10e-6, 10e-4), learning rate (0.01, 0.001, 0.0001), n_attention_heads (8, 16, 32), and dropouts (0, 0.1, 0.3, 0.5).

#### Message passing neural network (MPNN)

MPNN, first proposed by Gilmer and coworkers in 2017 [47], represents a commonly used GNN framework for various chemical prediction tasks. Many new GNN architectures have been developed based on the excellent performance and flexibility of MPNN framework for molecular property prediction [49, 73–75]. Herein, the main hyperparameters were optimized as follows: weight_decay (10e-8, 10e-6, 10e-4), learning rate (0.01, 0.001, 0.0001), graph_conv_layers ([64, 64], [128, 128], [256, 256]), num_layer_set2set (2, 3, 4), node_out_feats (16, 32, 64), and edge_hidden_feats (16, 32, 64).

#### Attentive FP

Attentive FP is an advanced GNN model that allows the model to focus on the most important elements of the input using graph attention mechanism [48]. It has been reported to exhibit SOTA performance for predicting molecular properties. Herein, the primary hyperparameters including dropout (0, 0.1, 0.5), graph feat size (50, 100, 200), num timesteps (1, 2, 3), num layers (2, 3, 4), learning rate (0.0001, 0.001, 0.01), and weight decay (0, 0.01, 0.0001), were optimized for the development of the Attentive FP models.

#### D-MPNN (Chemprop)

D-MPNN (Chemprop) was developed upon the MPNN framework by adopting a message-passing paradigm based on updating representations of directed bonds rather than atoms [49]. Chemprop has been successfully applied for the discovery of structurally distinct antibiotics [76]. Herein, the hyperparameters were optimized as follows: dropout (2, 3), dropout gat (0, 0.05), dim (1, 2), and gat scale (300, 400).

#### FP-GNN

Recently, FP-GNN as a novel DL architecture [50] was developed in our Lab for enhanced molecular properties prediction. FP-GNN not only learns to characterize the local atomic environment by propagating node information from nearby nodes to more distant nodes using the attention mechanism in a task-specific encoding, but also simultaneously learns a strong prior knowledge based on the fixed and complementary molecular fingerprints (MACCS, PubChem, and Pharmacophore ErG fingerprints). We used FP-GNN algorithm to build models for the kinase profiling prediction task. The hyperparameters were optimized as the following: dropout (0, 0.05, 0.1, 0.15, 0.2, 0.25, 0.3, 0.35, 0.4, 0.45, 0.5, 0.55, 0.6), dropout gat (0, 0.05, 0.1, 0.15, 0.2, 0.25, 0.3, 0.35, 0.4, 0.45, 0.5, 0.55, 0.6), dim (300, 350, 400, 450, 500, 550, 600), gat scale (0.2, 0.3, 0.4, 0.5, 0.6, 0.7, 0.8), nheads (2, 3, 4, 5, 6, 7, 8), and nhid (40, 45, 50, 55, 60, 65, 70, 75, 80).

The RF, SVM, KNN, and NB models were constructed using the Scikit-learn python package (https://github.com/scikit-learn/scikit-learn, version: 0.24.1) [77]; the XGBoost models were developed using the XGBoost python package (https://github.com/dmlc/xgboost, version: 1.3.3) [43]; four graph-based models (GCN, GAT, MPNN and Attentive FP) were established using the DeepChem python package (https://deepchem.io/); D-MPNN (Chemprop) models were constructed using the Chemprop python package (https://github.com/chemprop/chemprop); and FP-GNN models were developed using the FP-GNN software (https://github.com/idrugLab/FP-GNN). All ML and DL models were trained on CPU (Intel(R) Xeon(R) Silver 4216 CPU@2.10 GHz) and GPU (NVIDIA Corporation GV100GL [Tesla V100 PCIe 32 GB]), respectively. Additionally, Bayesian optimization was applied to optimize hyperparameters for FP-GNN and Chemprop models, while grid search method was employed to optimize hyperparameters for other models.

### Performance evaluation metric

To benchmark the performance of different ML and DL tools for the kinase profiling prediction, six metrics, including specificity (SP/TNR), sensitivity (SE/TPR/Recall), Balanced accuracy (BA), F1 score, Matthew’s correlation coefficient (MCC), and area under the receiver operating characteristic (ROC) curve (AUC), are used and defined as follows:

1 $$\mathrm{SP }=\frac{TN}{TN+FP}$$

2 $$\mathrm{SE }=\frac{TP}{TP+FN}$$

3 $$\mathrm{BA }=\frac{TPR+TNR}{2} = \frac{SE+SP}{2}$$

4 $${\text{F}}1 =\frac{2\times Precision\times Recall}{Precision+Recall}= \frac{2\times TP}{2\times TP+FN+FP}$$

5 $$\mathrm{MCC }=\frac{TP\times TN-FN\times FP}{\sqrt{\left(TP+FN\right)\times \left(TP+FP\right)\times \left(TN+FN\right)\times \left(TN+FP\right)}}$$

where TP, TN, FP, and FN represent the number of true positives, true negatives, false positives, and false negatives, respectively.

AUC was the most commonly used criterion for kinase inhibitor activity prediction tasks [15, 29, 30, 34, 35, 78], we therefore selected AUC value as the indicator of the accuracy of the classification models for a fair comparison. Given that active compounds outnumbered inactive compounds in the current kinase profiling modelling dataset, with a positive-to-negative ratio of 3.83, F1 score was also utilized to judge the accuracy of the models [34, 79–81].

## Results and discussion

### Benchmark dataset analysis and model construction

We obtained a comprehensive kinase profiling modelling dataset from multiple sources by applying the criteria mentioned in the Methods section. This dataset contains 141,086 unique molecules involving in 216,823 inhibitory activity data points, which covers 354 kinases from nine groups in the human kinome: TK family (88 kinases), CMGC family (48 kinases), AGC family (44 kinases), CAMK family (46 kinases), STE family (38 kinases), TKL family (30 kinases), Atypical family (16 kinases), CK1 family (6 kinases), and Others (38 kinases), Detailed information of the dataset are shown in Additional file 2: Table S1. The average ratio of positive (actives) to negative (inactives) was approximately 3.83, implying that the modelling dataset is relatively unbalanced. Nonetheless, in order to objectively explore and evaluate the predictive performance of different computational methods, we preferred to utilize the raw data from experimentally validated molecules against these kinases, without adding theoretical decoys to deliberately balance the modelling dataset. Bemis–Murcko scaffold analysis was conducted to analyze the structural diversity of molecules in the dataset. The proportion of scaffolds to molecules for each kinase falls between 10 and 100%, with an average value of 51.0%, suggesting that the molecules of the dataset were structurally diverse. Besides, compounds have broad distributions of molecular weight (36.461–998.013) and AlogP (-8.895–11.509), indicating that the compounds in the modelling dataset have an extensive chemical space (Additional file 2: Table S2). Such results imply that the predictive models based on this dataset could exhibit better reliability and robustness.

For this comprehensive kinase profiling modelling dataset, a total of 148,680 classification predictive models were generated based on the three different types of molecular features using the selected 12 ML and DL algorithms. To fairly compare the performance of the ML and DL methods for the kinase profiling predictive task, the average of the evaluation metrics of the established models for each algorithm were calculated as the final result. The details of performance of the established models are described and discussed in the following sections.

### Performance evaluation results of fingerprint-based ML and DL models

Five ML (KNN, NB, RF, SVM, and XGBoost) and one DL (DNN) approaches were used to build 106,200 predictive models based on five types of fingerprints (Morgan, MACCS, AtomPairs, FP2 and PharmacoPFP). Each model is denoted as a combination of the ML method and the corresponding molecular representation (e.g., DNN::Morgan).

**Fig. 2:**
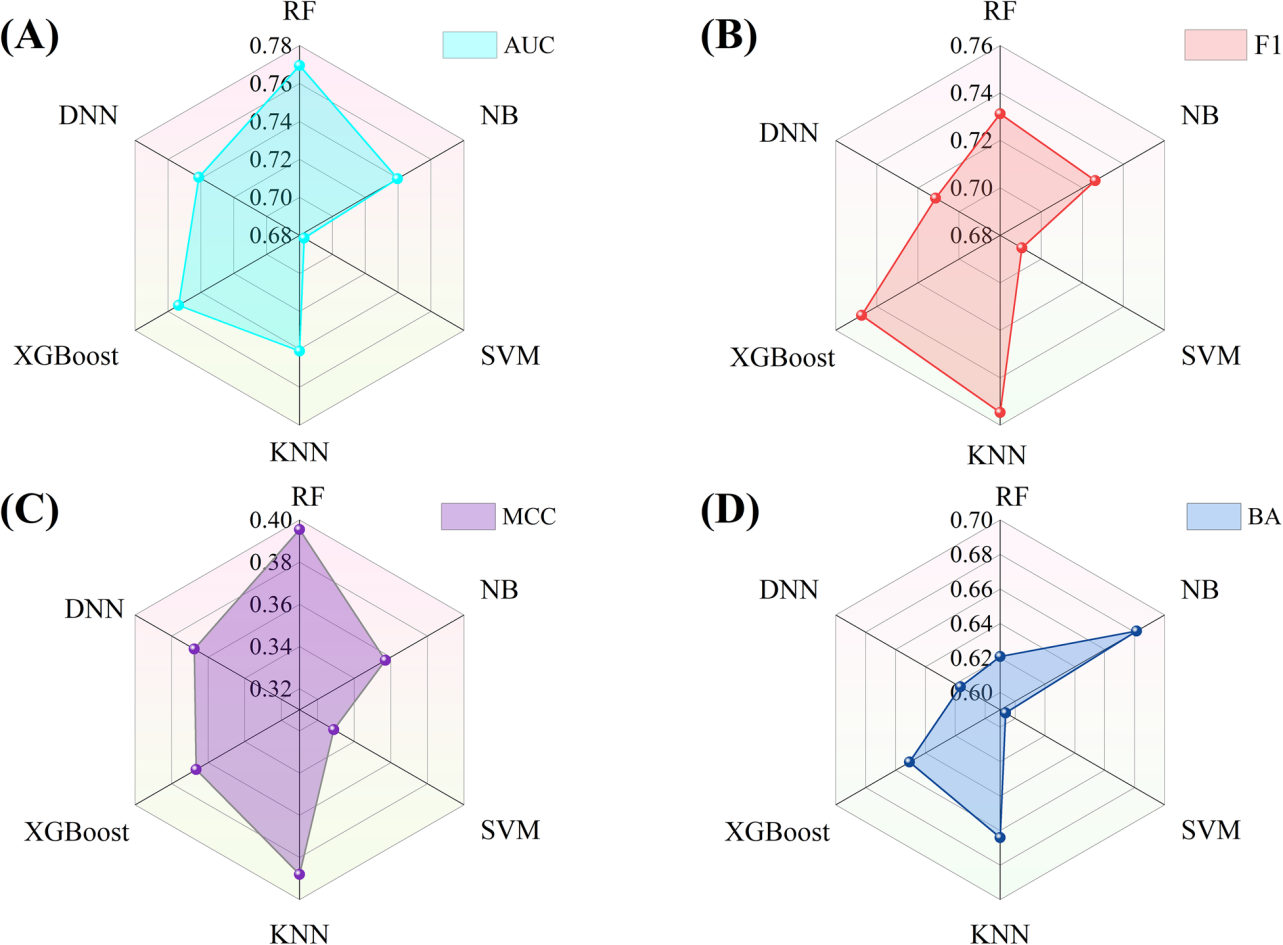
Performance comparison results of fingerprint-based models using different ML algorithms. **A**, **B**, **C** and **D** represent the comparison results based on the average F1 score, AUC, BA, and MCC values from the test sets, respectively

As shown in Table 2, most of the fingerprint-based models performed well for the kinase profiling predictive task, with an average AUC value > 0.73 and average F1 value > 0.72 on the test sets. Despite the differences in the characteristics of the five molecular fingerprints, the RF method performed the best for 354 kinases (Fig. 2), with the highest average AUC value (0.769) and MCC value (0.395), and relatively high F1 score (0.731) and BA value (0.621). In addition, another ensemble learning methods, XGBoost, also showed considerable predictive performance, achieving the second highest AUC value (0.754) and F1 score (0.747), and relatively high BA value (0.651) and MCC value (0.367).

**Table 2:** Performance comparison results of the fingerprint-based models on the test sets of 354 kinases

Molecular feature	Method	AUC ^*g*^	F1 score ^*h*^	BA ^*i*^
AtomPairs	RF^*a*^	0.779 ± 0.161	0.736 ± 0.259	0.625 ± 0.124
	NB^*b*^	0.733 ± 0.135	0.716 ± 0.186	0.680 ± 0.117
	SVM^*c*^	0.698 ± 0.214	0.712 ± 0.286	0.620 ± 0.157
	KNN^*d*^	0.743 ± 0.152	0.747 ± 0.222	0.665 ± 0.126
	XGBoost^*e*^	0.759 ± 0.167	0.750 ± 0.212	0.653 ± 0.127
	DNN^*f*^	0.752 ± 0.171	0.714 ± 0.238	0.631 ± 0.128
	Mean	0.744 ± 0.027	0.729 ± 0.017	0.646 ± 0.024
FP2	RF	0.786 ± 0.150	0.731 ± 0.258	0.634 ± 0.118
	NB	0.743 ± 0.141	0.728 ± 0.173	0.692 ± 0.121
	SVM	0.682 ± 0.259	0.686 ± 0.288	0.590 ± 0.191
	KNN	0.748 ± 0.149	0.760 ± 0.200	0.671 ± 0.121
	XGBoost	0.761 ± 0.163	0.752 ± 0.218	0.659 ± 0.125
	DNN	0.753 ± 0.179	0.722 ± 0.237	0.626 ± 0.132
	Mean	0.746 ± 0.035	0.730 ± 0.026	0.645 ± 0.036
MACCS	RF	0.751 ± 0.166	0.732 ± 0.257	0.613 ± 0.121
	NB	0.724 ± 0.142	0.720 ± 0.177	0.662 ± 0.117
	SVM	0.670 ± 0.253	0.681 ± 0.292	0.577 ± 0.190
	KNN	0.719 ± 0.147	0.750 ± 0.201	0.646 ± 0.119
	XGBoost	0.739 ± 0.168	0.741 ± 0.224	0.639 ± 0.124
	DNN	0.705 ± 0.181	0.697 ± 0.249	0.591 ± 0.121
	Mean	0.718 ± 0.028	0.720 ± 0.027	0.621 ± 0.033
Morgan	RF	0.774 ± 0.166	0.722 ± 0.282	0.612 ± 0.122
	NB	0.772 ± 0.143	0.745 ± 0.176	0.702 ± 0.124
	SVM	0.680 ± 0.268	0.685 ± 0.292	0.594 ± 0.192
	KNN	0.755 ± 0.154	0.755 ± 0.211	0.674 ± 0.124
	XGBoost	0.761 ± 0.164	0.749 ± 0.223	0.653 ± 0.128
	DNN	0.761 ± 0.176	0.715 ± 0.245	0.621 ± 0.132
	Mean	0.751 ± 0.035	0.729 ± 0.027	0.643 ± 0.041
PharmacoPFP	RF	0.757 ± 0.174	0.735 ± 0.258	0.620 ± 0.121
	NB	0.726 ± 0.144	0.722 ± 0.174	0.670 ± 0.123
	SVM	0.684 ± 0.240	0.689 ± 0.281	0.587 ± 0.184
	KNN	0.740 ± 0.147	0.761 ± 0.193	0.664 ± 0.120
	XGBoost	0.748 ± 0.175	0.745 ± 0.225	0.649 ± 0.129
	DNN	0.735 ± 0.183	0.709 ± 0.249	0.614 ± 0.130
	Mean	0.732 ± 0.026	0.727 ± 0.026	0.634 ± 0.032

^*a*^ RF: Random forest

^*b*^ NB: Naïve Bayesian

^*c*^ SVM: Support vector machine

^*d*^ KNN: K-Nearest Neighbor

^*e*^ XGBoost: Extreme gradient boosting

^*f*^ DNN: Deep neural networks

^*g*^ AUC: Area under the receiver operating characteristics curve

^*h*^ F1 scores: F1-measure

^*i*^ BA: Balanced accuracy. “ ± ” values represent standard deviations

The Morgan fingerprints achieved highest mean AUC value (0.751 ± 0.035, Table 2), which implies that it is a relatively better molecular representation for kinase profiling prediction. In addition, combining different ML methods and different molecular fingerprints yielded different performance results, indicating that it is necessary to screen the combination of modelling algorithms and feature expressions to achieve the best performance. For example, the RF and XGBoost algorithm tends to use the FP2 fingerprints as input features to achieve the best model rather than the Morgan fingerprints. In contrast, the NB algorithm tends to utilize the Morgan fingerprints as input features to generate the best models rather than the FP2 fingerprints (Table 2).

**Fig. 3:**
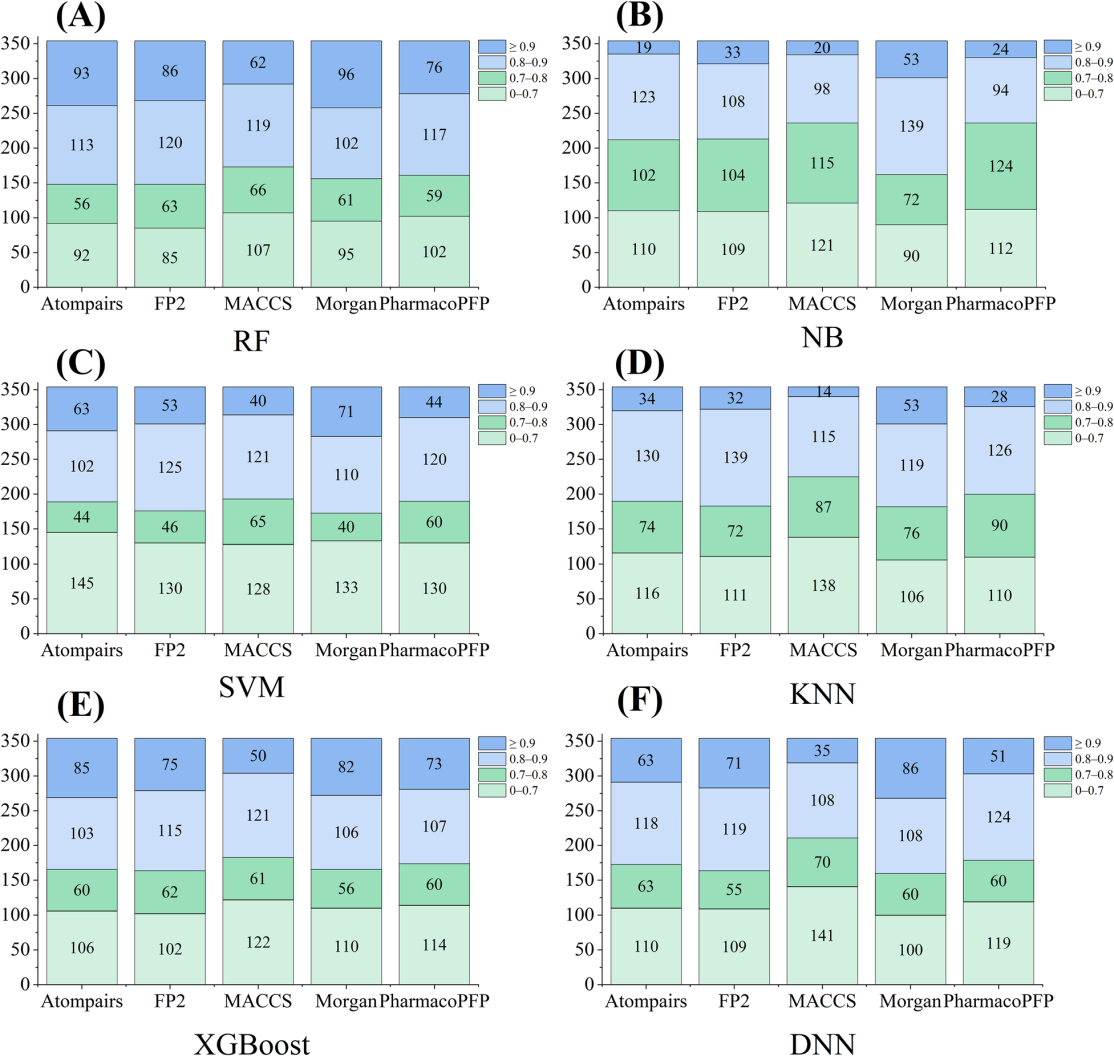
The interval distribution of the AUC values of fingerprint-based models for 354 kinases by using RF (**A**), NB (**B**), SVM (**C**), KNN (**D**), XGBoost (**E**), and DNN (**F**) algorithms

We further analyzed the interval distribution of the average AUC values of the test sets of 354 kinase targets for each method. As shown in Fig. 3, although different combinations of fingerprints and modelling methods can produce different distributions of AUC values, statistical analysis found that the AUC values ​​of the majority of the fingerprint-based models (~ 72.2%) were greater than 0.7. For example, the numbers of high quality (HQ, AUC > 0.7) for the RF::AtomPairs and XGBoost::AtomPairs models were 262 (Fig. 3A) and 248 (Fig. 3E) kinases, respectively. In addition, the RF::FP2 models showed obvious advantage, achieving the highest average AUC value (0.786 ± 0.150, Table 2). Importantly, it can achieve AUC values ​​greater than 0.7 on 269 kinases (Fig. 3A).

**Fig. 4:**
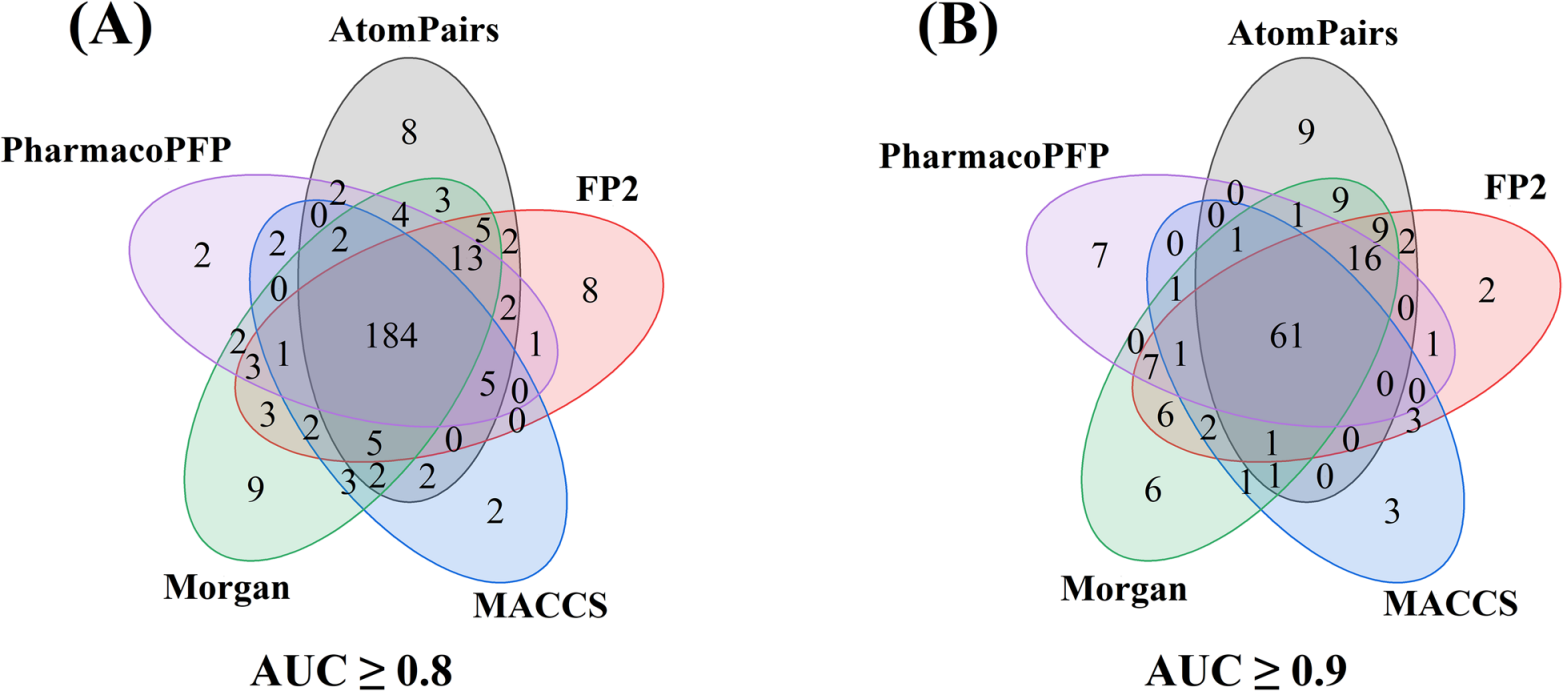
Overlap analyses of various fingerprint-based high-quality (HQ) models with an average AUC of ≥ 0.8 (**A**) and ≥ 0.9 (**B**), respectively

The Morgan fingerprints owns the relatively better predictive performance with highest average AUC value, however, this does not necessarily mean that other fingerprints cannot outperform the Morgan fingerprints on individual kinases. Figure 4A showed that the FP2, AtomPairs, MACCS, and PharmacoPFP fingerprints contributed eight, eight, two, and two unique kinase targets in the models with an AUC ≥ 0.8. Although the Morgan fingerprints also contributed the most models with an AUC ≥ 0.8, and the majority of these models were commonly found by at least two of other four fingerprints (i.e. FP2, MACCS, Morgan and PharmacoPFP fingerprints). The most unique HQ models was obtained by the AtomPairs fingerprints with an average AUC greater than 0.9 (Fig. 4B), i.e. the FP2, MACCS, Morgan and PharmacoPFP fingerprints can generates two, three, six, and seven unique HQ models that cannot be obtained by the AtomPairs fingerprints.

Recently, Merget et al. [30] reported RF models based Morgan fingerprints for the profiling prediction of kinase inhibitors, with an average AUC of 0.76 on 291 kinases, and achieving HQ (AUC > 0.7) on ~ 200 kinases. Apparently, the RF::FP2 models proposed in this study are superior to the models from Merget et al. study in terms of the total of number of kinases (354) and the overall accuracy (mean AUC = 0.786), as well as the number of HQ models (269, AUC > 0.7). In addition, the RF::Morgan models proposed herein have comparable or superior performance to the models of Merget et al., i.e. it exhibited average AUC value of 0.774 on 354 kinases and achieved HQ models on 259 kinases. The results illustrated that the comprehensive kinase profiling dataset with large structural diversity and chemical space constructed in this paper is necessary for building robust and reliable kinase profiling prediction models, as well as the optimal combination of ML algorithms and molecular feature representations can help to develop more accurate models for the virtual profiling prediction of kinase inhibitors.

### Performance evaluation results of descriptor-based ML and DL models

Subsequently, a total of 21,240 descriptor-based predictive models were successfully constructed and compared using the same modelling methods. The optimized RDKit-descriptors obtained using the SelectPercentile module (Percentile = 30) implemented in the scikit-learn package were utilized as input features for model construction. Detailed performance results of the descriptor-based models are listed in Additional file 2: Table S3. The average F1, AUC, and BA values for the test sets of these models are summarized in Table 3.

**Table 3:** Performance comparison results of RDKit descriptor-based predictive models on the test sets of 354 kinases

Molecular feature	Method	AUC^*g*^	F1 score^*h*^	BA^*i*^
RDKitDes	RF^*a*^	0.798 ± 0.120	0.759 ± 0.225	0.650 ± 0.113
	NB^*b*^	0.763 ± 0.099	0.739 ± 0.155	0.681 ± 0.090
	SVM^*c*^	0.727 ± 0.206	0.723 ± 0.245	0.611 ± 0.165
	KNN^*d*^	0.774 ± 0.116	0.776 ± 0.186	0.684 ± 0.104
	XGBoost^*e*^	0.755 ± 0.148	0.747 ± 0.216	0.650 ± 0.117
	DNN^*f*^	0.718 ± 0.180	0.693 ± 0.254	0.589 ± 0.117
	Mean	0.756 ± 0.030	0.740 ± 0.029	0.644 ± 0.038

^*a*^ RF: Random forest

^*b*^ NB: Naïve Bayesian

^*c*^ SVM: Support vector machine

^*d*^ KNN: K-Nearest Neighbor

^*e*^ XGBoost: Extreme gradient boosting

^*f*^ DNN: Deep neural networks

^*g*^ AUC: Area under the receiver operating characteristics curve

^*h*^ F1 scores: F1-measure

^*i*^ BA: Balanced accuracy. “ ± ” values represent standard deviations

As shown Table 3, most descriptor-based predictive models performed quite well, with mean F1 scores = 0.74, and average AUC value greater than 0.75. In accordance with the fingerprint-based models evaluation results where RF method achieved the best performance, RF::RDKitDes also performed best with the highest average AUC value (0.798 ± 0.120) (Table 3) on these descriptor-based models, which by the way is higher than any other fingerprint-based models (Table 2). According to the average AUC values of these descriptor-based models (Table 3), KNN method achieved the second-ranked predictive performance, followed by NB and XGBoost methods.

**Fig. 5:**
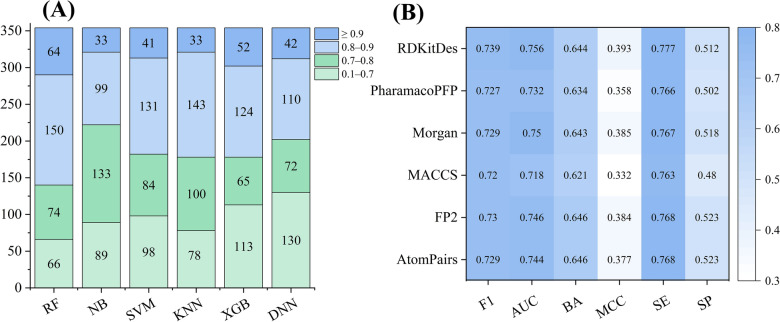
**A** Detailed distribution of the average AUC values of RDKitDes-based models for 354 kinases. **B** Heatmap analysis results of the average metrics of RDKitDes- and fingerprint-based models on the test sets

Figure 5A illustrates that approximately 73% of the descriptor-based models are HQ models, which outperform the aforementioned fingerprint-based models. Taking the RF::RDKitDes model as an example, it not only achieved the highest mean AUC value, but achieved 288 HQ models (Fig. 5A) for 354 kinases. Clearly, the RF::RDKitDes model outperforms the corresponding RF-based fingerprint models in terms of both the average AUC metric and the number of HQ models (Table 2 and Fig. 3A), regardless of which molecular fingerprints is used as input features.

To further confirm whether descriptor-based models outperform fingerprint-based models, we systematically compare the evaluation metrics of these models. As shown in Fig. 5B, RDKitDes-based models slightly outperformed fingerprint-based models due to their best performances in terms of the high average F1 score, AUC, SE and MCC values. The detailed comparison results of descriptor- and fingerprint-based models for each ML algorithm are shown in Additional file 1: Fig. S1. For example, RDKitDes-based models achieved the highest F1 scores and AUC values on the RF, SVM, and KNN algorithms (Additional file 1: Figs. S1A, C and D), and slightly weaker and/or comparable performance on the NB, XGBoost and DNN methods (Additional file 1: Figs. S1B, E and F), when compared to fingerprint models based on these ML algorithms. These results highlighted that RDKitDes may be suitable for achieving the optimal performance of ML methods in the kinase profiling prediction task.

### Performance evaluation results of graph-based DL models

**Fig. 6:**
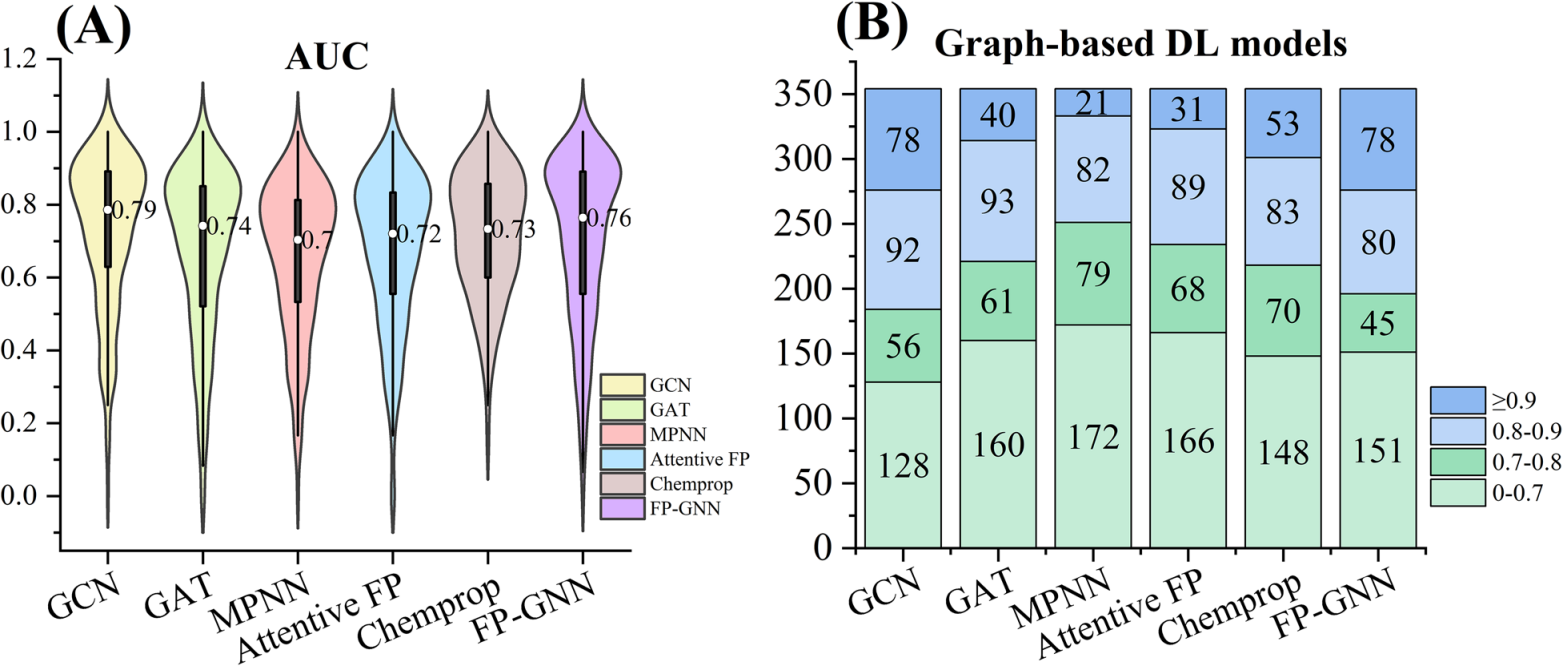
**A** Violin plot of the overall distribution of AUC values for six graph-based DL models. White spheres represent the medians, and boxes represents 1.5 the interquartile range (1.5IQR) from the median. **B** Detailed distribution of the average AUC values of different graph-based DL models on 354 kinases

Currently, various graph-based DL algorithms, which have recently been developed and achieved the SOTA performance in molecular property prediction tasks [48, 49, 82], have not been used for the kinase profiling prediction task. Accordingly, we introduced six GNN-based DL algorithms (Table 4) to model the kinase profiling prediction task. As shown in Table 4, GCN exhibited the overall best performance on the test sets compared to other GNN-based DL methods, achieving the highest average AUC (0.729 ± 0.206) and BA (0.604 ± 0.127) values, and second high F1 score (0.658 ± 0.271). A violin plot analysis of the overall AUC values also demonstrated that GCN performed the best (Fig. 6A), followed by FP-GNN and GAT methods.

**Table 4:** Performance comparison results of different graphs-based DL models on the test sets

Molecular feature	Method	AUC^*g*^	F1 score^*h*^	BA^*i*^
Molecular graphs	GCN^*a*^	0.729 ± 0.206	0.658 ± 0.271	0.604 ± 0.127
	GAT^*b*^	0.675 ± 0.225	0.636 ± 0.272	0.582 ± 0.145
	MPNN^*c*^	0.658 ± 0.202	0.621 ± 0.298	0.557 ± 0.128
	Attentive FP^*d*^	0.674 ± 0.207	0.661 ± 0.295	0.581 ± 0.116
	Chemprop^*e*^	0.717 ± 0.173	0.640 ± 0.291	0.573 ± 0.108
	FP-GNN^*f*^	0.704 ± 0.223	0.627 ± 0.367	0.604 ± 0.142
	Mean	0.693 ± 0.028	0.641 ± 0.016	0.584 ± 0.018

^*a*^ GCN: Graph convolutional network

^*b*^ GAT: Graph attention network

^*c*^ MPNN: Message passing neural networks

^*d*^ Attentive FP

^*e*^ Chemprop: D-MPNN

^*f*^ FP-GNN

^*g*^ AUC: Area under the receiver operating characteristics curve

^*h*^ F1 scores: F1-measure

^*i*^BA: Balanced accuracy. “ ± ” values represent standard deviations

Further analysis of the distribution of AUC values shows that the GCN models and FP-GNN models exhibited comparable performance in terms of HQ models, achieving 78 models in the interval where the AUC value is greater than 0.9 (Fig. 6B). Additionally, the GCN models and FP-GNN models, respectively, outperformed the RF::RDKitDes models on 140 and 143 kinases in terms of AUC metric (Additional file 2: Tables S4-S5. Consequently, the predictive models based on the GCN and FP-GNN algorithms are more applicable overall compared to other graph-based DL methods.

**Fig. 7:**
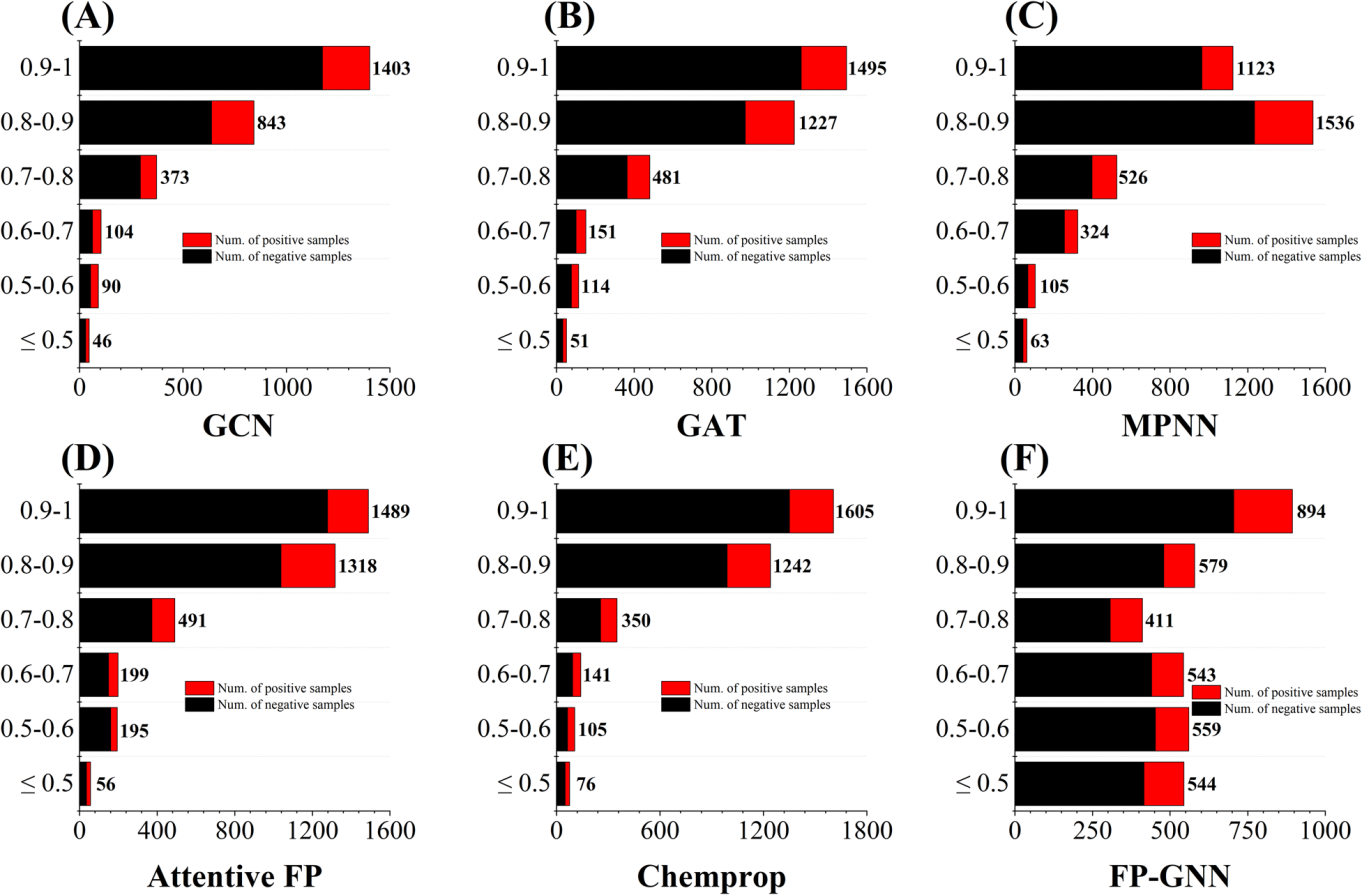
Relationships between the interval distribution of AUC values in the test sets and the corresponding interval of different compound quantities in the training sets of GCN (**A**), GAT (**B**), MPNN (**C**), Attentive FP (**D**), Chemprop (**E**), and FP-GNN (**F**) models

However, the use of graph-based DL methods (Table 4) may not be suitable as they do not show any advantage in the kinase profiling prediction task compared to the models based on the fixed prior molecular features such as molecular fingerprints (Table 2) and descriptors (Table 3). Even GCN and FP-GNN models only achieved 226 and 203 HQ models (AUC > 0.7) for 354 kinase targets. Typically, graph-based DL algorithms have an inherent self-learning mechanism, which may result in poor performance due to the insufficient modelling datasets in individual kinases. To confirm this point, we further analyze whether the size of the modelling dataset for each kinase has an impact on the accuracy of the graph-based DL models. Figure 7 summarizes the relationship between the AUC values in the test sets and compound quantity intervals in the training sets for the graph-based DL models. In general, the prediction performance is positively correlated with the number of compounds in the training set. Taking the GCN method as an example (Fig. 7A), if the number of molecules in modelling dataset is less than 100, few HQ models can be obtained. Similar phenomena are observed in other DL methods (Figs. 7B–F), albeit with some differences. In other words, graph-based DL models possibly acquire better predictive performance on large datasets. Our findings further illustrate the shortcomings of graph-based DL algorithms in the field of kinase prediction, especially for kinases with insufficient activity data. In the future, as the number of kinases and their inhibitors continues to increase, graph-based DL algorithms may be more suitable for many individual kinases to achieve better predictive performance.

### Comparison performance results of fingerprint-, descriptor-, and graph-based ML and DL models

**Fig. 8:**
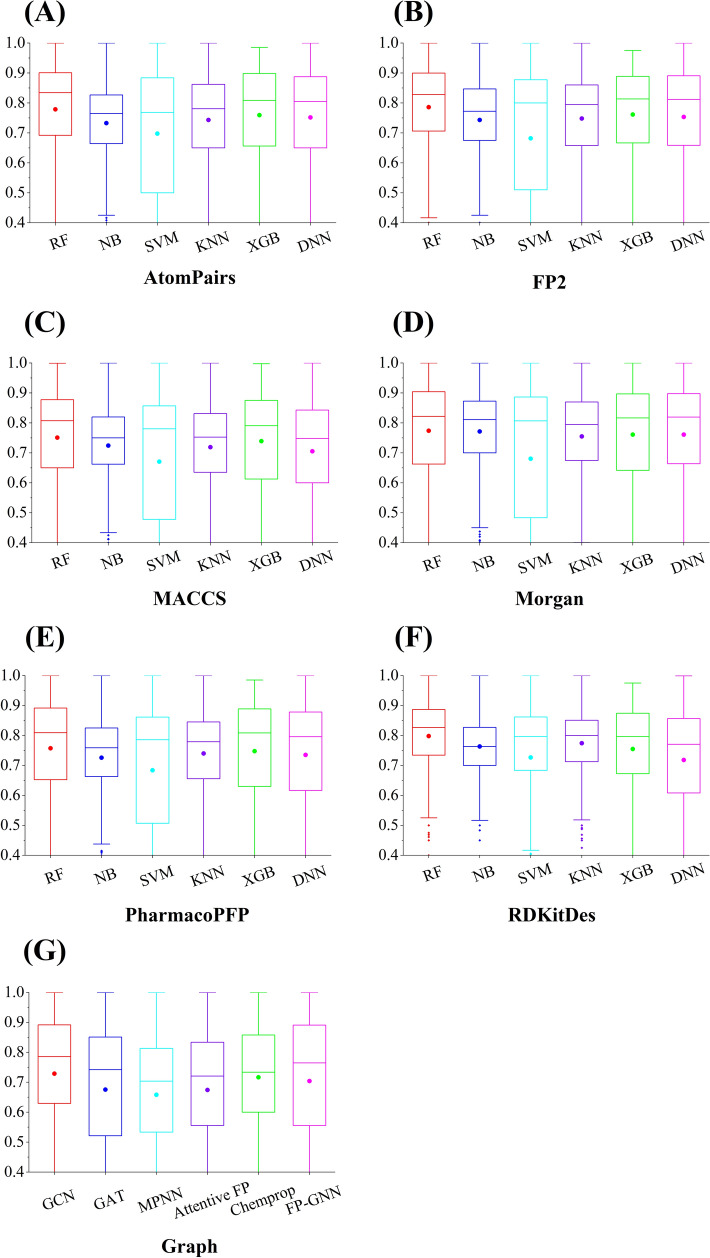
Comparison of average AUC values of **A** AtomPairs-, **B** FP2-, **C** MACCS-, **D** Morgan-, **E** PharmacoPFP-, and **F** RDKitDes-, **G** Graph-based models using five ML and one DNN DL methods. The average AUC values of the test sets for various ML and DL algorithms are displayed as boxplot. Middle spheres represent the median, and boxes represent the interquartile range (IQR) from the median

Boxplots analysis for AUC values of descriptor-, fingerprint-, and graph-based models on the test sets are shown in Fig. 8. If considering the commonly used AUC value as the final evaluation metric, the RF::RDKitDes models (Fig. 8F) performed best, followed by RF::FP2, RF::AtomPairs, and RF::Morgan models. It is clear that RF method usually achieved the best performance (Fig. 8) for the kinase profiling prediction task when molecular descriptors and fingerprints are used as input features. When F1 score, BA and MCC values were used as the final assessment metric (Additional file 1: Figs. S2-S4), RF also showed comparable performance. In addition, the average predictive performance of graph-based DL algorithms (Fig. 8G and Figs. S2G-S4G) are inferior to fingerprints- and descriptor-based ML models. The optimal in silico predictive models for each kinase in terms of AUC metric are shown in Additional file 2: Table S6.

For better comparison of the predictive performance of deep learning to a variety of other prediction methods, based on KinaseNet dataset, we added multi-task GCN, GAT, DNN, FP-GNN, Chemprop and Attentive FP models. A total of six deep learning methods were adopted to construct the corresponding multi-task deep learning models, and hyperparameters optimazation were performed to stretch the ability of algorithms. As shown in Table 5, compared with single model, multi-task learning can promote the comprehensive prediction ability of the model, and improve the prediction ability of models on the multi-task data set. In addition, the multi-task FP-GNN model achieves the highest average AUC of 0.807, which is higher than the best descriptor models (0.798) and fingerprint models (0.786). Besides, the multi-task FP-GNN model’s performance is close to but slightly worse than RF::AtomPairs + FP2 + RDKitDes fusion model (0.825). These results show that the effects of descriptor-based and graph-based models vary from data set to data set. Although current research focuses on graph-based multitask modeling strategies, and many graph-based deep learning and multi-task models claim to have the most advanced performance in predictive tasks, there is still much debate about the performance of algorithms based on molecular fingerprints and descriptors versus those based on molecular pictures and structures.

**Table 5:** Performance of AUC values based on multi-task models

Single models	AUROC	Multi-task models	AUROC
GCN	0.729	Multi-GCN	0.785
GAT	0.675	Multi-GAT	0.713
FP-GNN	0.704	FP-GNN	0.807
Chemprop	0.717	Chemprop	0.798
AttentiveFP	0.674	AttentiveFP	0.667
MPNN	0.658	Multi-DNN::Morgan	0.556

### Exploring whether combining descriptors and fingerprints could improve the performance of models

To investigate whether the combined features of fingerprints and descriptors could improve the performance of the kinase profiling prediction task, the combined features were used to establish 10,620 models using six ML algorithms. As shown in Table 6, the combined-features-models based on the RF, XGBoost and DNN algorithms slightly outperformed their corresponding descriptor- and fingerprint-based models in terms of AUC metric. For example, the best combined-features-model (RF::Morgan::RDkitDes, AUC = 0.815, Table 6) is superior to RF::RDKitDes and RF::Morgan. Similar trends occurred in the comparative performance of the combined-features-models and individual descriptor- and fingerprint-based models in terms of F1 score (Additional file 2: Table S7). However, the predictive performance of the combined-features-models constructed using KNN, NB, and SVM methods did not outperform the corresponding descriptor-based models (Table 6, because the average AUC values of these combined models were slightly larger than that of the fingerprint-based models, but smaller than that of the descriptor-based models. A possible reason is that more input of feature information is conducive to building accurate prediction models for the ensemble learning RF and XGB algorithms and DNN method.

**Table 6:** Performance comparison results of AUC values between the combined-features-based models and individual descriptor- and fingerprint-based models

Method	Combined features	AUC	Molecular feature	AUC	Difference
DNN^*a*^	AtomPairs::RDKitDes	0.749	AtomPairs	0.752	− 0.003
	FP2::RDKitDes	0.762	FP2	0.753	0.009
	MACCS::RDKitDes	0.741	MACCS	0.705	0.036
	Morgan::RDKitDes	0.774	Morgan	0.761	0.013
	PharamacoPFP::RDKitDes	0.748	PharamacoPFP	0.735	0.013
			RDKitDes	0.718	
KNN^*b*^	AtomPairs::RDKitDes	0.745	AtomPairs	0.743	0.002
	FP2::RDKitDes	0.754	FP2	0.748	0.006
	MACCS::RDKitDes	0.742	MACCS	0.719	0.023
	Morgan::RDKitDes	0.767	Morgan	0.755	0.012
	PharmacoPFP::RDKitDes	0.749	PharmacoPFP	0.740	0.009
			RDKitDes	0.774	
NB^*c*^	AtomPairs::RDKitDes	0.738	AtomPairs	0.733	0.005
	FP2::RDKitDes	0.747	FP2	0.743	0.004
	MACCS::RDKitDes	0.750	MACCS	0.724	0.026
	Morgan::RDKitDes	0.781	Morgan	0.772	0.009
	PharmacoPFP::RDKitDes	0.737	PharmacoPFP	0.726	0.011
			RDKitDes	0.763	
RF^*d*^	AtomPairs::RDKitDes	0.792	AtomPairs	0.779	0.013
	FP2::RDKitDes	0.803	FP2	0.786	0.017
	MACCS::RDKitDes	0.799	MACCS	0.751	0.048
	Morgan::RDKitDes	0.815	Morgan	0.774	0.041
	PharmacoPFP::RDKitDes	0.801	PharmacoPFP	0.757	0.044
			RDKitDes	0.798	
SVM^*e*^	AtomPairs::RDKitDes	0.699	AtomPairs	0.698	0.001
	FP2::RDKitDes	0.686	FP2	0.682	0.004
	MACCS::RDKitDes	0.681	MACCS	0.670	0.011
	Morgan::RDKitDes	0.685	Morgan	0.680	0.005
	PharmacoPFP::RDKitDes	0.687	PharmacoPFP	0.684	0.003
			RDKitDes	0.727	
XGBoost^*f*^	AtomPairs::RDKitDes	0.763	AtomPairs	0.759	0.004
	FP2::RDKitDes	0.768	FP2	0.761	0.007
	MACCS::RDKitDes	0.758	MACCS	0.739	0.019
	Morgan::RDKitDes	0.768	Morgan	0.761	0.007
	PharmacoPFP::RDKitDes	0.763	PharmacoPFP	0.748	0.015
			RDKitDes	0.755	

^*a*^ DNN: Deep neural networks

^*b*^ KNN: K-Nearest Neighbor

^*c*^ NB: Naïve Bayesian

^*d*^ RF: Random forest

^*e*^ SVM: Support vector machine

^*f*^XGBoost: Extreme gradient boosting

### Exploring whether model fusion could improve performance on the kinase profiling prediction task

**Fig. 9:**
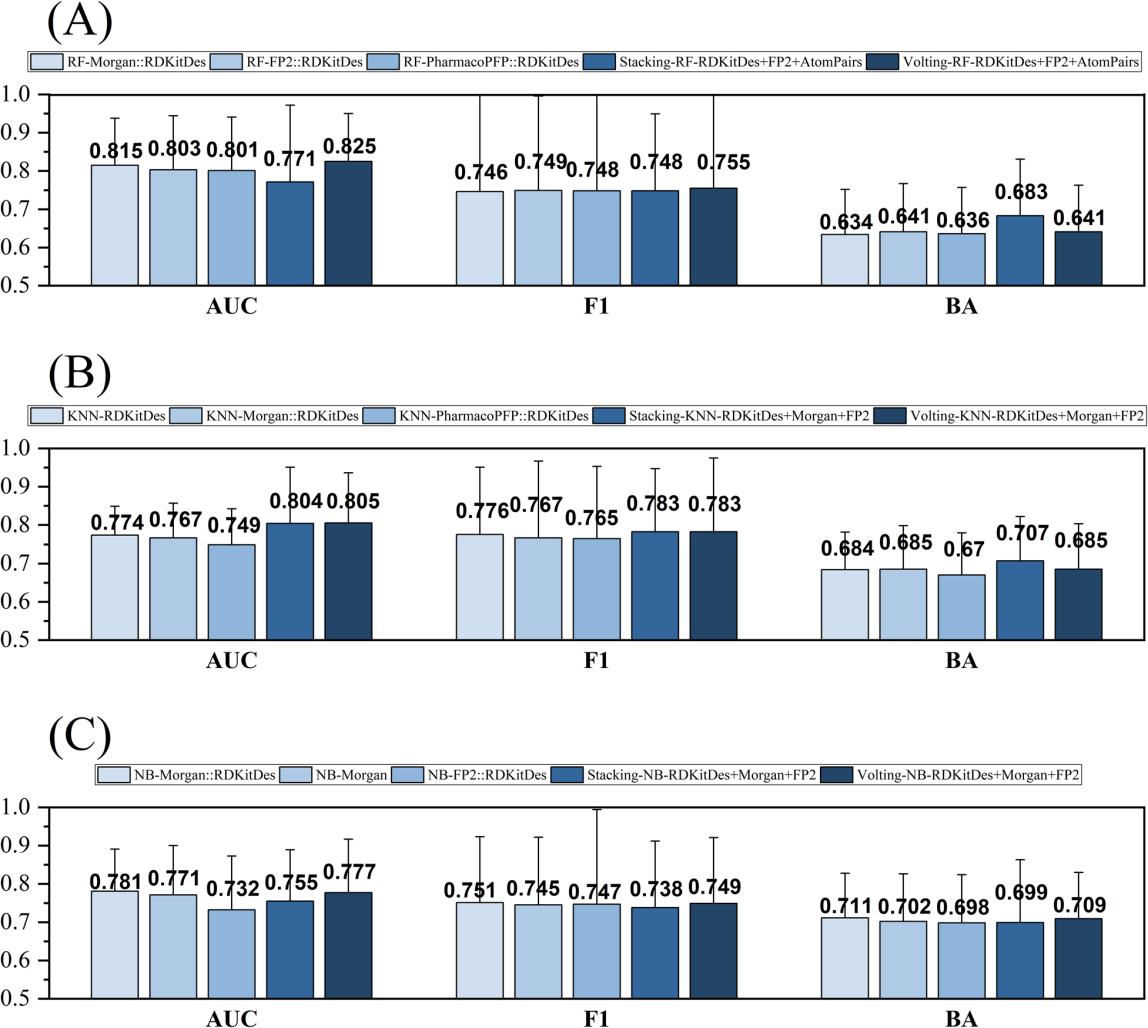
Comparison of the prediction results between fusion models and single models. The fusion models are constructed based on RF (**A**), KNN (**B**), and NB (**C**) models using voting and stacking methods

We further explore whether fusion models can improve classification accuracy of a single model in the kinase profiling prediction task. Given that the RF, KNN and NB algorithms outperformed other ML and DL methods on the kinase profiling prediction task (Additional file 2: Table S8), both voting and stacking methods were therefore used construct fusion model based on the three ML algorithms. As shown in Fig. 9, both voting- and stacking-based fusion models were slightly better than the corresponding single-based RF and KNN models, albeit with some differences in terms of NB models. For example, the voting fusion models based on RF achieved the best overall performance with the highest average values of AUC (0.825 ± 0.124).

**Fig. 10:**
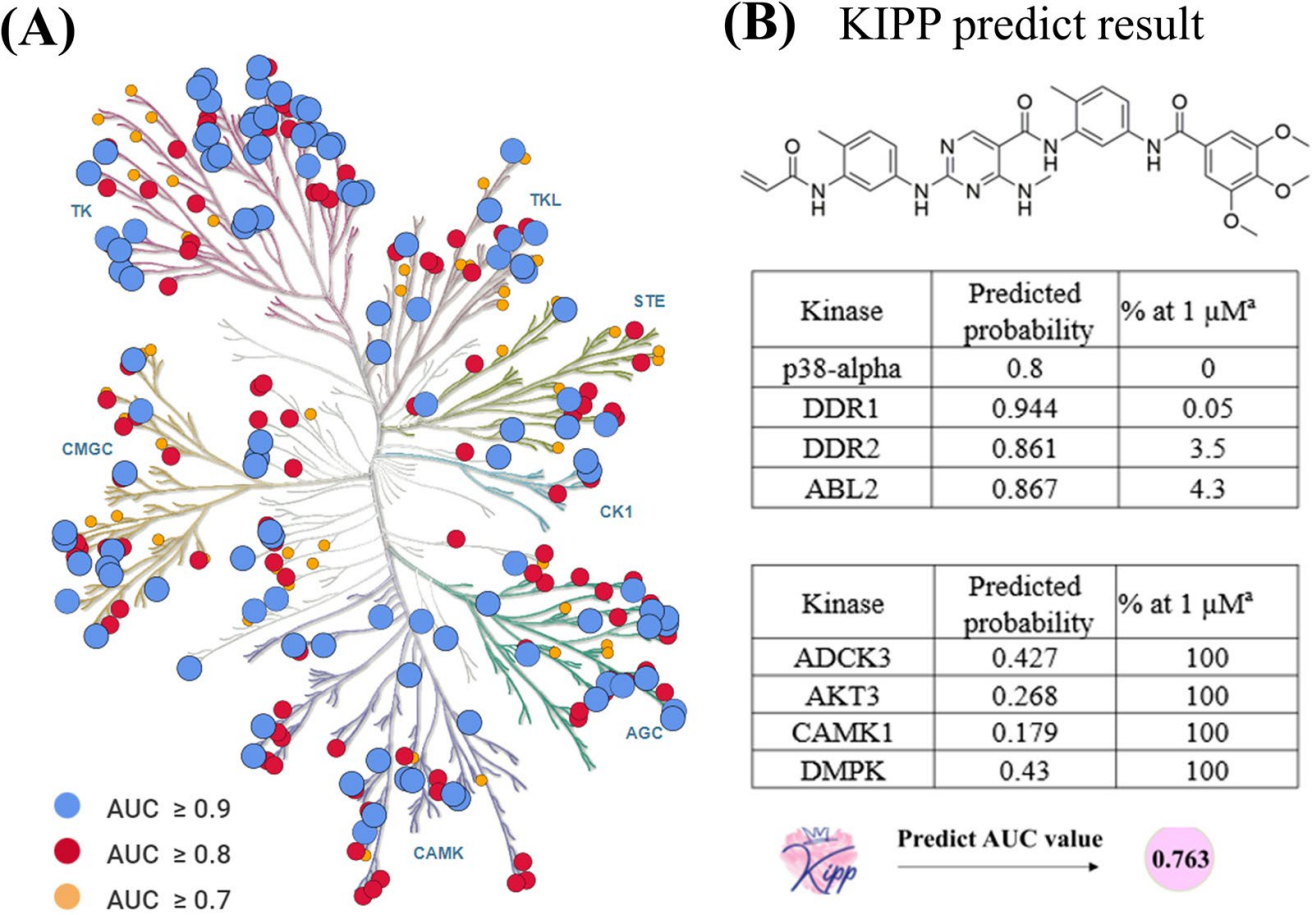
**A** Kinome map analysis of the RF::AtomPairs + FP2 + RDKitDes models. Kinases are colored based on their AUC values. The kinase tree was generated using Kinmap tool (http://kinhub.org/kinmap) [83]. **B** Chemical structure of CHMFL-BMX-078 and its predicted result. AUC value (0.763) was generated based on the predicted kinase profile of CHMFL-BMX-078 using KIPP and its experimentally tested kinase profile. BMX: bone marrow kinase in the X chromosome

Collectively, RF::AtomPairs + FP2 + RDKitDes voting fusion models achieved the overall best performance in the kinome-wide profiling prediction task in terms of AUC metric. As shown in Fig. 10A, 301 HQ models were obtained in the voting fusion models and distributed over the entire kinome tree covering all kinase families.

### KIPP online webserver construction and application

Although several kinases profiling prediction models have been reported (Additional file 2: Table S9), easy-to-use software and/or online webserver are not available. To this end, an online platform called KIPP (https://kipp.idruglab.cn/) was developed based on the overall optimal RF::AtomPairs + FP2 + RDKitDes models (default). A collection of the best models based on each kinase and the multi-task FP-GNN model are also provided. KIPP includes five main modules: compound basic information display, kinase profiling prediction and display, kinase tree construction and display, selectivity index calculation and display, and similarity search results display. Overall selectivity and selectivity towards a kinase subfamily will be generated based on the predicted kinase profile. The overall selectivity is represented by the two quantitative evaluation methods, standard score [84] and Gini coefficient [85]. Odds ratio (OR) is adopted to calculate sub-family selectivity to represent the strength of the association between an inhibitor and a sub-family [86].

**Fig. 11:**
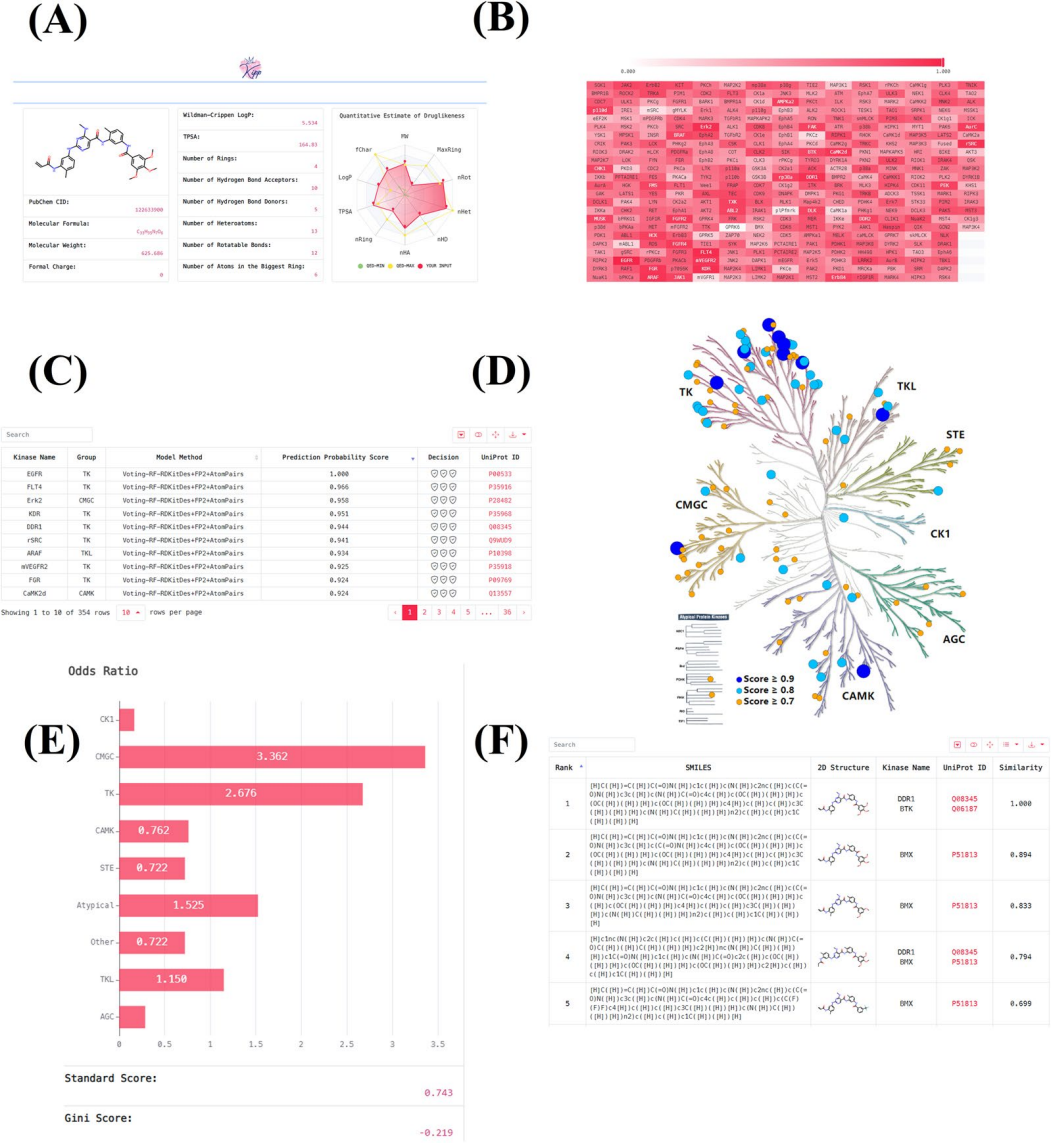
Website schematic diagram of KIPP for CHMFL-BMX-078 in the kinase profiling prediction task. **A** represents the basic information for submitted CHMFL-BMX-078. **B** and **C** represent kinase profiling prediction results of CHMFL-BMX-078 in heat map and list, respectively. **D** represents kinase tree diagram of CHMFL-BMX-078. **E** represents the selectivity index results. **F** represents the similarity search results

Taking CHMFL-BMX-078 (a highly potent and selective Type II irreversible BMX kinase inhibitor) [87] as an example, users can easily upload the SMILES or draw the structure online of CHMFL-BMX-078 (Fig. 10B) to quickly predict the inhibitory activity of this compound against 354 kinase across the kinome. Once the calculation task is completed, users can click on different modules to query the calculation results, including basic compound information (Fig. 11A), kinase profiling prediction results in heat map (Fig. 11B) and list (Fig. 11C), kinase tree diagram (Fig. 11D), selectivity index results (Fig. 11E) and similarity search results for the CHMFL-BMX-078 (Fig. 11F). The predicted kinase profiling results of CHMFL-BMX-078 by KIPP were overall consistent with the experimental kinases inhibition results (Additional file 2: Table S10), with an AUC value of 0.763, indicating the accuracy and usability of the KIPP platform. Importantly, native versions of Python software are also provided for various kinases, allowing users to perform large-scale VS.

## Conclusions

In this paper, we provided a comprehensive assessment of the performance of five ML (NB, RF, XGBoost, KNN, and SVM) and seven DL (DNN, GCN, GAT, MPNN, D-MPNN, Attentive FP, and FP-GNN) methods in kinase profiling prediction task. To obtain a more objective performance evaluation, we constructed a comprehensive KinaseNet dataset covering 354 kinases cross the entire kinome to benchmark all tools. Three types of commonly used molecular features, including a set of molecular descriptors, a collection of five molecular fingerprints (Morgan, MACCS keys, AtomParis, FP2, and PharmacoPFP), and molecular graphs, were used as input features to build predictive models using these compared methods. We found that RF outperform the other methods for kinase profiling prediction. This finding generalizes across different types of molecular descriptors and fingerprints. Meanwhile, the RDKitDes-based models generally outperform fingerprint-based models. Specifically, the RF::RDKitDes models performed best, followed by RF::FP2, RF::AtomPairs, and RF::Morgan models. Although single-task graph-based DL methods do not achieve the best overall predictive performance on the KinaseNet dataset, the predictive performance of multi-task DL models such as multitask FP-GNN and Chemprop models can still achieve comparable or even better predictive performance than conventional descriptor- and fingerprint-based models, due to the existence of certain data linkages between the various kinase data. In addition, these performance of DL methods improves as the training dataset increases. Accordingly, we envision that with the increasing amounts and quality of data from industry and academia, further performance improvements could be gained by DL methods. Combining descriptors and fingerprints could improve the performance of models, especially for the fingerprint-based models. In addition, fusion models based on the voting and stacking methods further improve performance on the kinase profiling prediction task. Finally, an easy-to-use online platform KIPP and its local version software were constructed based on the optimal models for various kinase inhibitor identification related tasks, including kinase profiling prediction, virtual screening, drug repositioning, and target fishing. It is expected that this study can provide valuable guidance for researchers who are interested in developing innovative and even more powerful kinase profiling prediction models, as well as for medicinal chemists and pharmacologists in designing and discovering new kinase inhibitors.

### Supplementary Information

**Additional file 1: Fig S1.** Detailed comparison performance of descriptor- and fingerprint-based models using various ML algorithms. (A), (B), (C), (D), (E), and (F) represent the comparison results for the RF, NB, SVM, KNN, XGB, and DNN methods, respectively. **Fig S2.** Comparison of average F1 scores of (A) AtomPairs-, (B) FP2-, (C) MACCS-, (D) Morgen-, (E) PharmacoPFP-, (F) RDKitDes-, and (G) Graph-based models. The assay-F1 scores for various ML algorithms are displayed as boxplot. Middle spheres represent the median, and boxes represents the interquartile range (IQR) from the median. **Fig S3.** Comparison of average BA values of (A) AtomPairs-, (B) FP2-, (C) MACCS-, (D) Morgen-, (E) PharmacoPFP-, (F) RDKitDes-, and (G) Graph-based models. The assay-BA values for various ML algorithms are displayed as boxplot. Middle spheres represent the median, and boxes represent the interquartile range (IQR) from the median. **Fig S4.** Comparison of average MCC values of (A) AtomPairs-, (B) FP2-, (C) MACCS-, (D) Morgen-, (E) PharmacoPFP-, and (F) RDKitDes-, (G) Graph-based models. The assay-MCC values for various ML algorithms are displayed as boxplot. Middle spheres represent the median, and boxes represent the interquartile range (IQR) from the median.

**Additional file 2: Table S1.** Details on benchmark dataset for kinase profiling prediction task used in this study. **Table S2.** Structural diversity and chemical space analysis of the compounds in each kinase. **Table S3.** Detailed performance results of different ML methods. **Table S4.** Detailed individual kinases where the GCN models outperform the RF::RDKitDes models. **Table S5.** Detailed individual kinases where the FP-GNN models outperform the RF::RDKitDes models. **Table S6.** The optimal in silico predictive models for each kinase in terms of AUC metric. **Table S7.** Comparison performance of models based on combined features and single feature in terms of F1 score. **Table S8.** Ranking of all single models by AUC values. **Table S9.** Comparison of our models with the reported in silico prediction models for kinase profiling prediction task. **Table S10.** The predicted activity probability and experimental % activity of CHMFL-BMX-078.

## Data Availability

KIPP online platform is freely accessible at https://kipp.idruglab.cn/. Datasets and python version executable software of KIPP are freely available on Github: https://github.com/idrugLab/KinasePredictPro.
