# Supplementary material for: Large-scale comparison of machine learning methods for profiling prediction of kinase inhibitors
Source: J Cheminform. 2024 Jan 30;16:13. doi: 10.1186/s13321-023-00799-5 (PMC10829268; doi:10.1186/s13321-023-00799-5)
Supplement: Supplementary file 1 — Additional file 1: Fig S1. Detailed comparison performance of descriptor- and fingerprint-based models using various ML algorithms. (A), (B), (C), (D), (E), and (F) represent the comparison results for the RF, NB, SVM, KNN, XGB, and DNN methods, respectively. Fig S2. Comparison of average F1 scores of (A) AtomPairs-, (B) FP2-, (C) MACCS-, (D) Morgen-, (E) PharmacoPFP-, (F) RDKitDes-, and (G) Graph-based models. The assay-F1 scores for various ML algorithms are displayed as boxplot. Middle spheres represent the median, and boxes represents the interquartile range (IQR) from the median. Fig S3. Comparison of average BA values of (A) AtomPairs-, (B) FP2-, (C) MACCS-, (D) Morgen-, (E) PharmacoPFP-, (F) RDKitDes-, and (G) Graph-based models. The assay-BA values for various ML algorithms are displayed as boxplot. Middle spheres represent the median, and boxes represent the interquartile range (IQR) from the median. Fig S4. Comparison of average MCC values of (A) AtomPairs-, (B) FP2-, (C) MACCS-, (D) Morgen-, (E) PharmacoPFP-, and (F) RDKitDes-, (G) Graph-based models. The assay-MCC values for various ML algorithms are displayed as boxplot. Middle spheres represent the median, and boxes represent the interquartile range (IQR) from the median. [file 13321_2023_799_MOESM1_ESM.docx]

**Additional Information**

**Large-scale comparison of machine learning methods for profiling prediction of kinase inhibitors**

Jiangxia Wu‡, Yihao Chen‡, Jingxing Wu‡, Duancheng Zhao, Jindi Huang,MuJie Lin, Ling Wang*,†

^a^Guangdong Provincial Key Laboratory of Fermentation and Enzyme Engineering, Joint International Research Laboratory of Synthetic Biology and Medicine, Guangdong Provincial Engineering and Technology Research Center of Biopharmaceuticals, School of Biology and Biological Engineering, South China University of Technology, Guangzhou 510006, China.

*E-mail: [lingwang@scut.edu.cn](mailto:lingwang@scut.edu.cn)

**Supplementary Figures and Tables**

**Fig S1.** Detailed comparison performance of descriptor- and fingerprint-based models using various ML algorithms. (A), (B), (C), (D), (E), and (F) represent the comparison results for the RF, NB, SVM, KNN, XGB, and DNN methods, respectively.

**Fig S2.** Comparison of average F1 scores of (A) AtomPairs-, (B) FP2-, (C) MACCS-, (D) Morgen-, (E) PharmacoPFP-, (F) RDKitDes-, and (G) Graph-based models. The assay-F1 scores for various ML algorithms are displayed as boxplot. Middle spheres represent the median, and boxes represents the interquartile range (IQR) from the median.

**Fig S3.** Comparison of average BA values of (A) AtomPairs-, (B) FP2-, (C) MACCS-, (D) Morgen-, (E) PharmacoPFP-, (F) RDKitDes-, and (G) Graph-based models. The assay-BA values for various ML algorithms are displayed as boxplot. Middle spheres represent the median, and boxes represent the interquartile range (IQR) from the median.

**Fig S4.** Comparison of average MCC values of (A) AtomPairs-, (B) FP2-, (C) MACCS-, (D) Morgen-, (E) PharmacoPFP-, and (F) RDKitDes-, (G) Graph-based models. The assay-MCC values for various ML algorithms are displayed as boxplot. Middle spheres represent the median, and boxes represent the interquartile range (IQR) from the median.

**Table S1.** Details on benchmark dataset for kinase profiling prediction task used in this study.

**Table S2.** Structural diversity and chemical space analysis of the compounds in each kinase.

**Table S3.** Detailed performance results of different ML methods.

**Table S4.** Detailed individual kinases where the GCN models outperform the RF::RDKitDes models.

**Table S5.** Detailed individual kinases where the FP-GNN models outperform the RF::RDKitDes models.

**Table S6.** The optimal in silico predictive models for each kinase in terms of AUC metric.

**Table S7.** Comparison performance of models based on combined features and single feature in terms of F1 score.

**Table S8.** Ranking of all single models by AUC values.

**Table S9.** Comparison of our models with the reported in silico prediction models for kinase profiling prediction task.

**Table S10.** The predicted activity probability and experimental % activity of CHMFL-BMX-078.


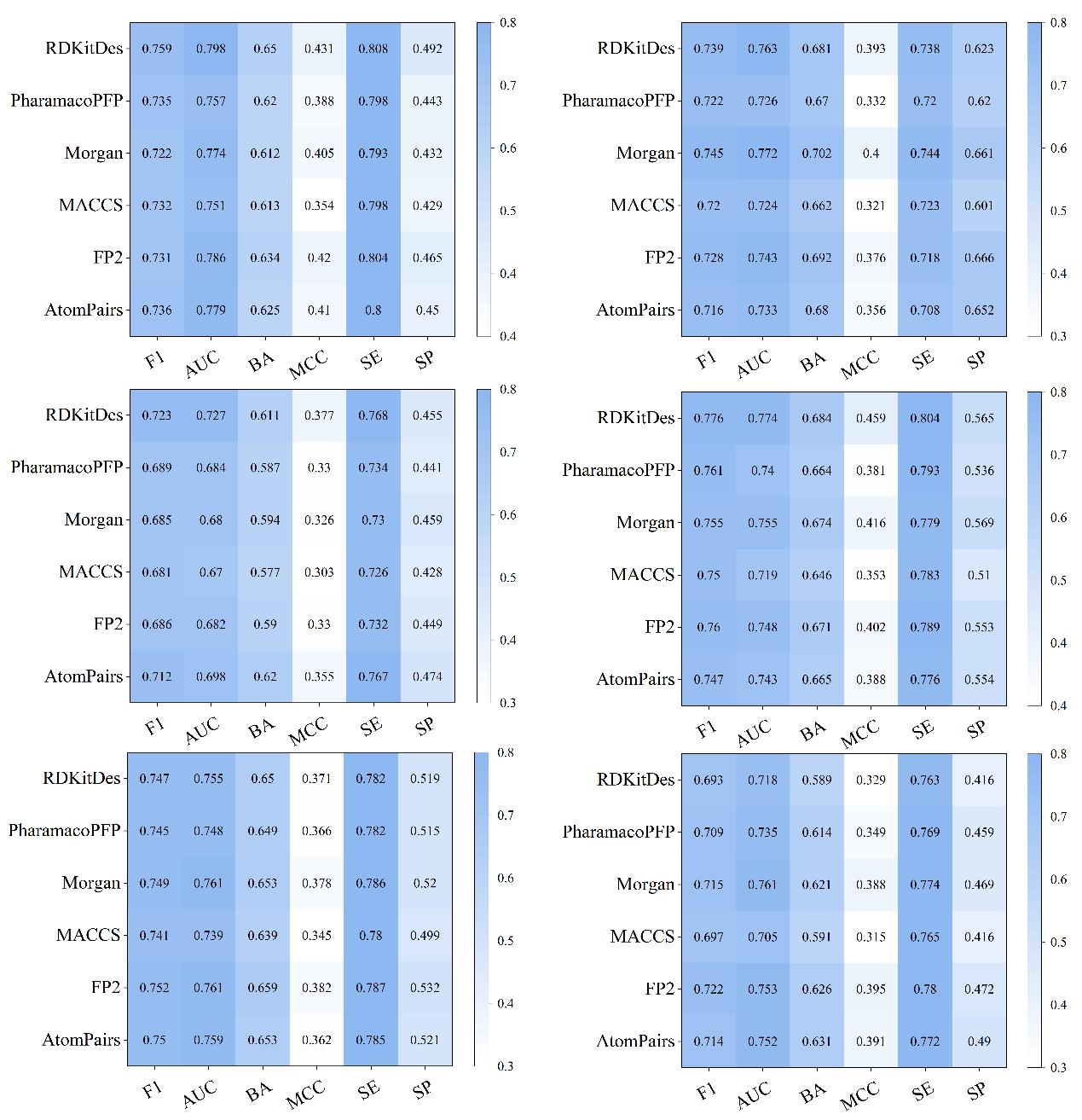


**Fig S1.** Detailed comparison performance of descriptor- and fingerprint-based models using various ML algorithms. (A), (B), (C), (D), (E), and (F) represent the comparison results for the RF, NB, SVM, KNN, XGB, and DNN methods, respectively.


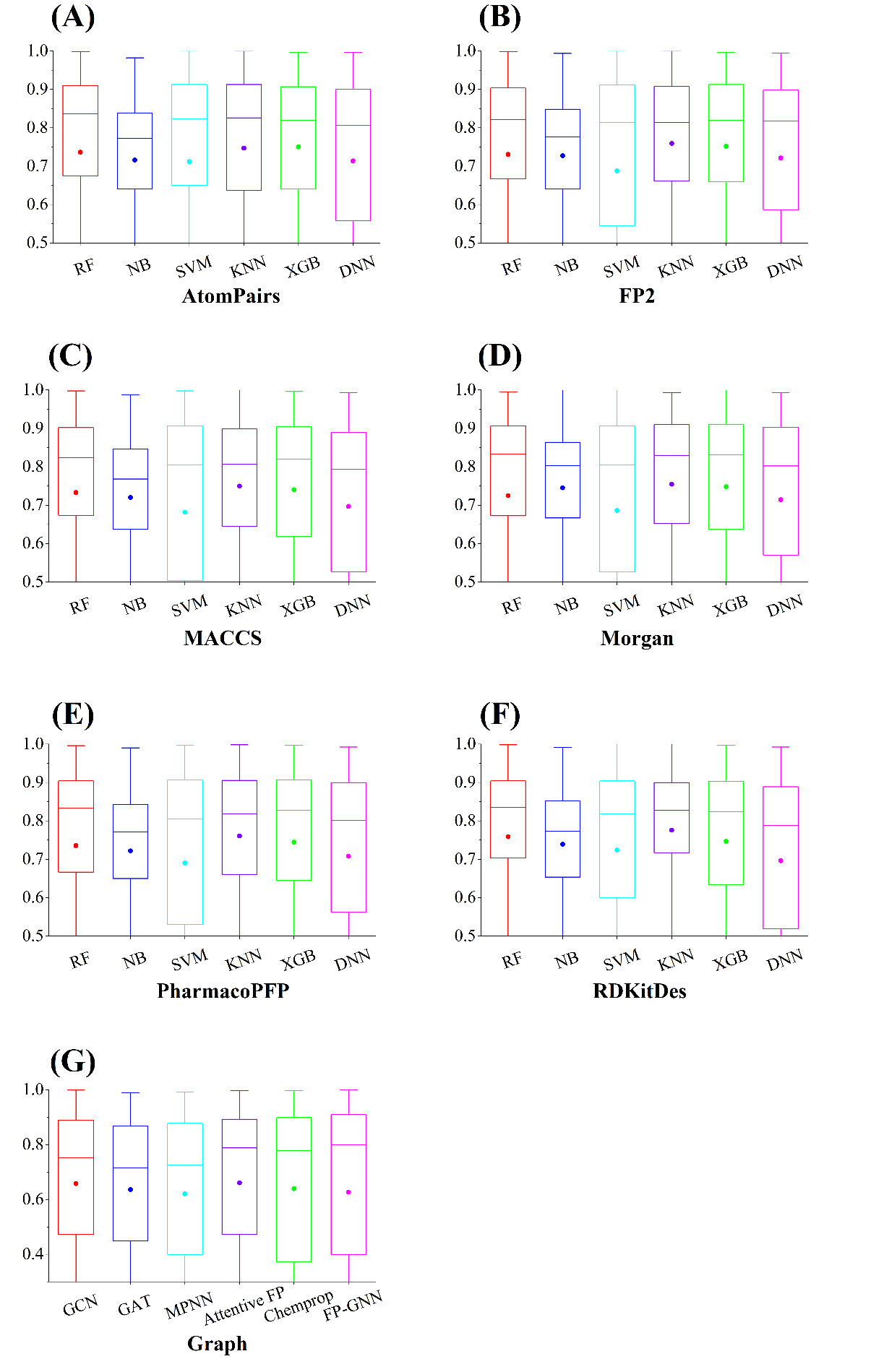


**Fig S2.** Comparison of average F1 scores of (A) AtomPairs-, (B) FP2-, (C) MACCS-, (D) Morgen-, (E) PharmacoPFP-, (F) RDKitDes-, and (G) Graph-based models. The assay-F1 scores for various ML algorithms are displayed as boxplot. Middle spheres represent the median, and boxes represents the interquartile range (IQR) from the median.


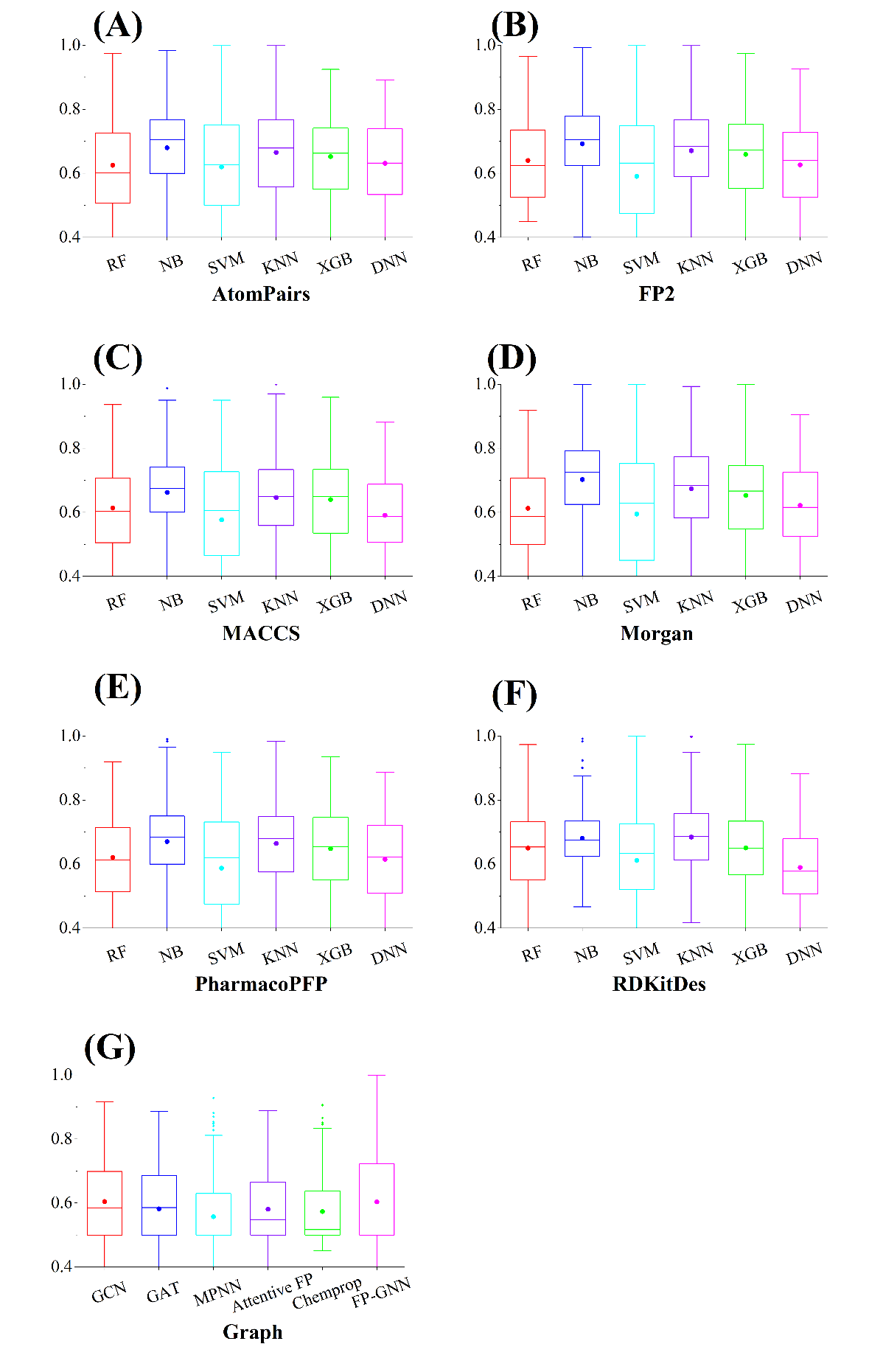


**Fig S3.** Comparison of average BA values of (A) AtomPairs-, (B) FP2-, (C) MACCS-, (D) Morgen-, (E) PharmacoPFP-, (F) RDKitDes-, and (G) Graph-based models. The assay-BA values for various ML algorithms are displayed as boxplot. Middle spheres represent the median, and boxes represent the interquartile range (IQR) from the median.


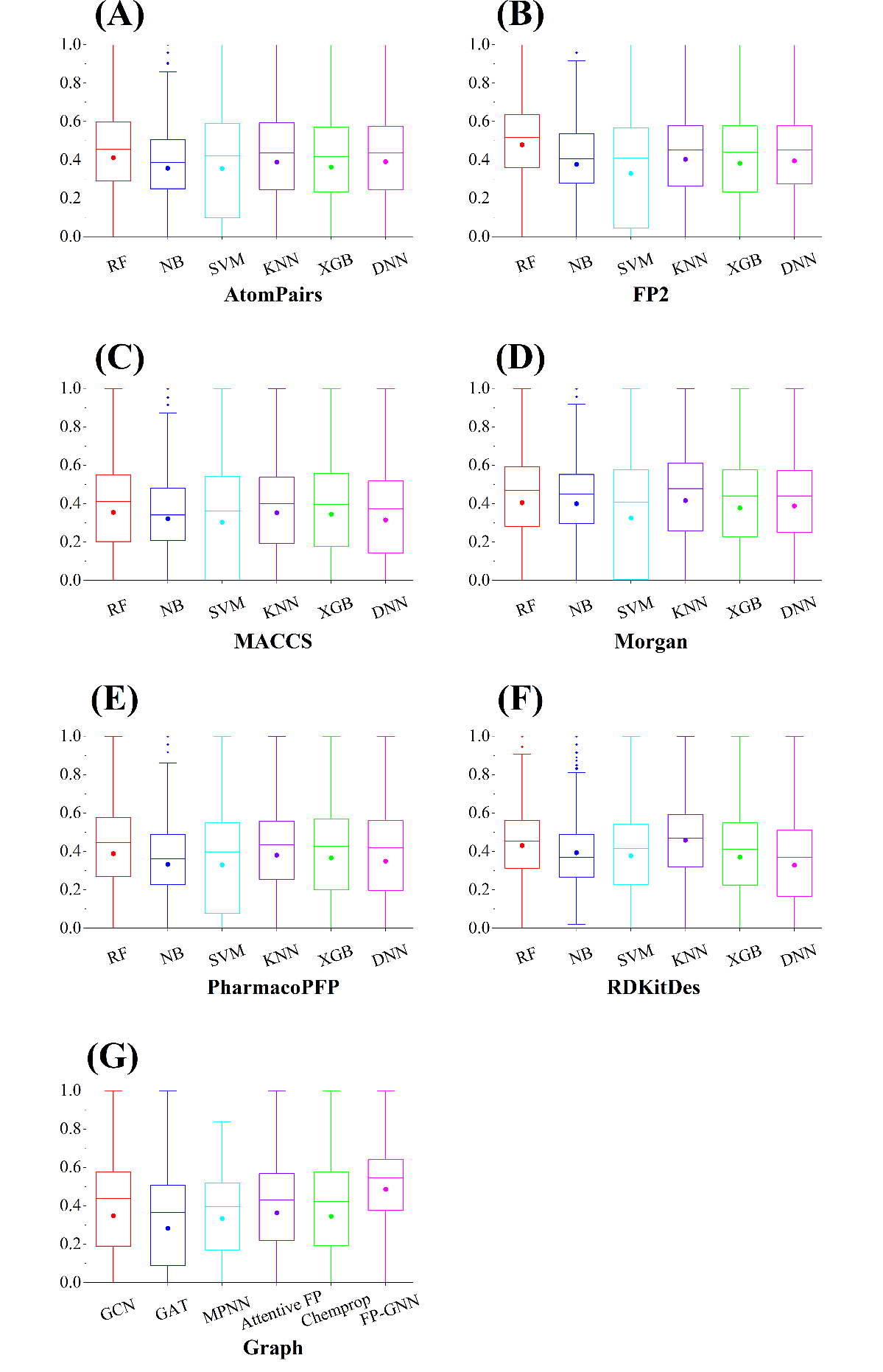


**Fig S4.** Comparison of average MCC values of (A) AtomPairs-, (B) FP2-, (C) MACCS-, (D) Morgen-, (E) PharmacoPFP-, and (F) RDKitDes-, (G) Graph-based models. The assay-MCC values for various ML algorithms are displayed as boxplot. Middle spheres represent the median, and boxes represent the interquartile range (IQR) from the median.
